# ZIP11 Regulates Nuclear Zinc Homeostasis in HeLa Cells and Is Required for Proliferation and Establishment of the Carcinogenic Phenotype

**DOI:** 10.3389/fcell.2022.895433

**Published:** 2022-07-11

**Authors:** Monserrat Olea-Flores, Julia Kan, Alyssa Carlson, Sabriya A. Syed, Cat McCann, Varsha Mondal, Cecily Szady, Heather M. Ricker, Amy McQueen, Juan G. Navea, Leslie A. Caromile, Teresita Padilla-Benavides

**Affiliations:** ^1^ Department of Molecular Biology and Biochemistry, Wesleyan University, Middletown, CT, United States; ^2^ Department of Biochemistry and Molecular Biotechnology, University of Massachusetts Chan Medical School, Worcester, MA, United States; ^3^ Department of Chemistry, Skidmore College, Saratoga Springs, NY, United States; ^4^ Department of Cell Biology, Center for Vascular Biology, UCONN Health-Center, Farmington, CT, United States

**Keywords:** ZIP11, zinc transport, cell cycle, senescence, gene expression, MTF1, cervical cancer cells

## Abstract

Zinc (Zn) is an essential trace element that plays a key role in several biological processes, including transcription, signaling, and catalysis. A subcellular network of transporters ensures adequate distribution of Zn to facilitate homeostasis. Among these are a family of importers, the Zrt/Irt-like proteins (ZIP), which consists of 14 members (ZIP1-ZIP14) that mobilize Zn from the extracellular domain and organelles into the cytosol. Expression of these transporters varies among tissues and during developmental stages, and their distribution at various cellular locations is essential for defining the net cellular Zn transport. Normally, the ion is bound to proteins or sequestered in organelles and vesicles. However, though research has focused on Zn internalization in mammalian cells, little is known about Zn mobilization within organelles, including within the nuclei under both normal and pathological conditions. Analyses from stomach and colon tissues isolated from mouse suggested that ZIP11 is the only ZIP transporter localized to the nucleus of mammalian cells, yet no clear cellular role has been attributed to this protein. We hypothesized that ZIP11 is essential to maintaining nuclear Zn homeostasis in mammalian cells. To test this, we utilized HeLa cells, as research in humans correlated elevated expression of ZIP11 with poor prognosis in cervical cancer patients. We stably knocked down ZIP11 in HeLa cancer cells and investigated the effect of Zn dysregulation *in vitro*. Our data show that ZIP11 knockdown (KD) reduced HeLa cells proliferation due to nuclear accumulation of Zn. RNA-seq analyses revealed that genes related to angiogenesis, apoptosis, mRNA metabolism, and signaling pathways are dysregulated. Although the KD cells undergoing nuclear Zn stress can activate the homeostasis response by MTF1 and MT1, the RNA-seq analyses showed that only ZIP14 (an importer expressed on the plasma membrane and endocytic vesicles) is mildly induced, which may explain the sensitivity to elevated levels of extracellular Zn. Consequently, ZIP11 KD HeLa cells have impaired migration, invasive properties and decreased mitochondrial potential. Furthermore, KD of ZIP11 delayed cell cycle progression and rendered an enhanced senescent state in HeLa cells, pointing to a novel mechanism whereby maintenance of nuclear Zn homeostasis is essential for cancer progression.

## Introduction

Zinc (Zn) is among the most abundant trace elements essential for life. As a micronutrient, Zn is involved in many biological processes, such as cell signaling, transcriptional modulation, and as a catalytic cofactor and structural component of several proteins (Reviewed by (Wu and Wu, 1987; Kambe et al., 2015)). Under physiological conditions, Zn is present in a non-redox active form as a divalent cation (Zn^2+^). Zn homeostasis plays a key role in human health, as Zn deficiencies have been identified as leading causes of diverse diseases. Patients lacking this ion may present skin abnormalities, hypogonadism, anemia, growth delays, alopecia, chronic inflammation, as well as deficiencies in immune, hepatic, and mental functions (Vallee and Falchuk, 1993; Hambidge, 2000; Maret and Sandstead, 2006; Devirgiliis et al., 2007; Takeda and Tamano, 2009; Sandstead, 2013). On the other hand, excess Zn is toxic and may disrupt the cellular acquisition of other micronutrients, such as copper (Cu) (Ogiso et al., 1979; Fischer et al., 1981; Broun et al., 1990). Total cellular Zn concentrations are typically in or below the micromolar range (Palmiter and Findley, 1995; Krezel and Maret, 2006; Colvin et al., 2008; Paskavitz et al., 2018; Gordon et al., 2019a; Gordon et al., 2019b; Tavera-Montañez et al., 2019). In general, 50% of subcellular Zn is located in the cytoplasm, 30–40% in the nucleus, and approximately 10% in the plasma membrane (Thiers and Vallee, 1957; Haase and Rink, 2014). However, Zn distribution may change depending on the developmental stage of the cells in a lineage-specific manner (Gordon et al., 2019a). The levels of labile, or “free,” Zn in the cytosol are low, ranging from picomolar and low nanomolar concentrations, as it is normally bound to proteins and sequestered into organelles and vesicles (Tavera-Montañez et al., 2019; Gordon et al., 2019a; Gordon et al., 2019b; Outten and O'Halloran, 2001; Qin et al., 2011; Vinkenborg et al., 2009; Sensi et al., 1997). To maintain low levels and adequate subcellular distribution of the ion, cells have developed complex systems to maintain Zn homeostasis.

Two families of Zn transporters mobilize Zn between the extracellular milieu, the cytoplasm, and the organelles (Dufner-Beattie et al., 2003a; Eide, 2006; Kambe et al., 2014; Kambe et al., 2015). The Zn transporter family (also named ZnT, solute-linked carrier 30, or SLC30) mediates cellular Zn export, while the Zrt- and Irt-like proteins (also named ZIP, solute-linked carrier 39, or SLC39) mediate cellular Zn import. ZnTs and ZIPs are transmembrane proteins with six or eight predicted transmembrane (TM) domains, respectively (Dufner-Beattie et al., 2003a; Kambe et al., 2015). Mammalian cells express nine ZnT (1–8, 10) exporters and 14 ZIP importers (1–14), but their contributions to Zn physiology continue to be largely understudied. These transporters maintain cytosolic Zn pools by mobilizing the ion from the extracellular space and intracellular compartments, as they are differentially distributed based on the cellular demands for Zn and stage of life (Lichten and Cousins, 2009; Jeong and Eide, 2013). The majority of ZIP transporters have a dynamic localization to the cell membrane, as their expression, internalization, and degradation is dependent on the levels of the ion (Chowanadisai et al., 2013; Weaver et al., 2007; Hojyo et al., 2011; Dufner-Beattie et al., 2003b; Kelleher and Lönnerdal, 2003; Liuzzi et al., 2004; Liu et al., 2008; Lichten et al., 2011; Gaither and Eide, 2000; Wang et al., 2004; Huang and Kirschke, 2007; Mao et al., 2007; Taylor et al., 2005). Though they mainly mobilize Zn, ZIP importers can also transport iron (Fe) (Liuzzi et al., 2006; Gao et al., 2008; Jenkitkasemwong et al., 2015), manganese (Mn), and cadmium (Cd) (Girijashanker et al., 2008; Fujishiro et al., 2012; Jenkitkasemwong et al., 2012; Gordon et al., 2019b).

To date, the only crystal structure available for a ZIP transporter is from the bacteria *Bordetella bronchiseptica* (BbZIP), which was obtained in the presence of Cd^2+^ (Zhang et al., 2017). BbZIP structure shows eight TM helices that are proposed to form a tight bundle. TM2, TM4, TM5, and TM7 constitute an inner bundle surrounded by the remaining TMs (Zhang et al., 2017). The BbZIP TM2 contains a 36 amino acid-long domain with a kink associated with a conserved proline (P110) (Zhang et al., 2017). TM4 and TM5 are also bent due to the presence of two proline residues in the metal-binding sites (MBS (Zhang et al., 2017)). BbZIP was found to have a novel symmetric structure. The first three TMs, TM1-TM3, are symmetrically related with the last three, TM6-TM8, by a pseudo-two-fold axis, which was defined to be almost parallel to the proposed membrane plane. Further, TM4 and TM5 also seem to be symmetrically related by the same axis, however these two segments appear to be fitted-in by the other two named 3-TM repeats (Zhang et al., 2017). This previously unrecognized architecture was defined as an unusual 3+2+3TM structure (Zhang et al., 2017). Crystallization of BbZIP in the presence of CdCl_2_ allowed for the identification of four Cd^2+^-binding sites and revealed that the amino- and carboxy-termini both face the extracellular domain (Zhang et al., 2017). This novel structural data support previous hydrophobicity plot predictions that suggested that ZIP transporters have eight TM helices with extracellular amino- and carboxy-terminal domains (Lichten and Cousins, 2009; Jeong and Eide, 2013).

To transport Zn, ZIP importers are proposed to form homodimers (Lin et al., 2010; Bin et al., 2011). Biochemical characterization and overexpression analyses have demonstrated that the apparent K_m_ ranges from hundreds of nM to approximately 20 µM (Gaither and Eide, 2000; Gaither and Eide, 2001a; Dufner-Beattie et al., 2003a; Wang et al., 2004; Liu et al., 2008; Pinilla-Tenas et al., 2011; Antala and Dempski, 2012; Dempski, 2012). Although the mechanism of Zn transport is not fully understood, early biochemical analyses of BbZIP suggested that Zn transport occurs in a channel-like, non-saturable electrogenic manner (Lin et al., 2010), and that phosphorylation by casein kinase 2 may also activate transport (Taylor et al., 2012). BbZIP crystallization points to a putative mechanism of Zn transport that may apply to other members of the ZIP family. Essentially, two conserved metal-binding residues, D113 and D305, seem to be necessary to recruit the metal to the transporter (Zhang et al., 2017). A conserved serine (S106) located at the bottom of the entrance cavity seems to be required to guide the ion into the transport pathway, while A102 was proposed to be a pore-lining residue at the extracellular side (Zhang et al., 2017). An inward-open conformation of the transporter can be stabilized by substrate binding at the binuclear metal center, which is in the middle of the transport pathway. Then the ion may be released to the cytoplasm through a “chain” of metal-binding residues (H177, E276, H275, and D144) and a histidine-rich loop that connects TM3 and TM4 (Zhang et al., 2017). These weak Zn-binding sites are located at the exit cavity and were named as a “metal sink,” proposed to facilitate metal release from the binuclear metal center (Zhang et al., 2017). Zn release is thought to occur due to the effect of repulsive electrostatic forces between the MBS and/or the removal of geometric constraints in the rearrangement of the TMs to form an open channel at the extracellular side of the membrane which may be blocked by conserved hydrophobic residues (M99 and A102 on TM2, L200 and I204 on TM5, and M269 on TM7) as the transporter opens to the cytosol (Zhang et al., 2017).

ZIP transporters are classified into subfamilies I, II, LIV-1, and GufA according to their sequence similarities (Taylor, 2000; Gaither and Eide, 2001b; Taylor and Nicholson, 2003; Yu et al., 2013; Hu, 2021). These transporters localize to specific cellular compartments and are regulated depending on cellular needs and stage of development or disease (Reviewed by (Kambe et al., 2015)). However, there is still a gap in our knowledge on the specific functions of some members of the family, such as ZIP11. This transporter was classified as a member of the GufA subfamily of ZIP proteins. The *ZIP11* gene contains several metal responsive elements (MRE), which are targets of the classic Metal Regulatory Transcription Factor 1 (MTF1) that enable *ZIP11* expression to respond to metal levels (Martin et al., 2013; Yu et al., 2013). However, it seems that this transporter is not largely induced by MTF1 upon increase in Zn levels, as are other transporters. In mice, a modest increase in *Zip11* mRNA expression was detected in the intestine and other organs (e.g. spleen) of animals exposed to acute oral Zn exposure (Yu et al., 2013). Thus, it was proposed that ZIP11 is not required to maintain the net quota of cellular Zn, and rather instead helps to maintain appropriate subcellular distribution of the ion. Gene expression analyses showed that the murine *Zip11* (*mZip11*) is highly expressed in the testes, stomach, ileum, and cecum, with a lower level of expression detected in the liver, duodenum, jejunum, and colon (Martin et al., 2013; Yu et al., 2013). Martin and coworkers (Martin et al., 2013) showed that within the murine gastrointestinal tract, ZIP11 is modestly downregulated by Zn deficiency in the stomach. This data showed that Zn deficiency may trigger the absorption of Z from the colon by ZIP4 rather than by ZIP11 (Martin et al., 2013).

Overexpression analyses determined that HEK cells expressing *mZip11-Flag* had elevated Zn content compared to controls. Moreover, incubation of cells expressing *mZip11-Flag* in the presence of Zn led to cell death after 2 days, while supplementation with the chelator N,N,N′N′-tetrakis (−) [2-pyridylmethyl]-ethylenediamine (TPEN) favored cell growth (Yu et al., 2013). Knockdown (KD) experiments in Raw264.7 cells consistently showed a decrease in cellular Zn levels, strengthening the hypothesis that mZip11 is a Zn importer (Yu et al., 2013). However, experiments using MDCK cells expressing the *mZip11-Flag* construct determined that the transporter may also mobilize Cu (Yu et al., 2013). Murine models have demonstrated that *Zip11* expression in different tissues have differential responses to Zn acquired from the diet (Martin et al., 2013; Yu et al., 2013). At the cellular level, ZIP11 is proposed to be localized to the nucleus and Golgi apparatus (Kelleher et al., 2012; Martin et al., 2013). Despite this evidence, the physiological and cellular functions of ZIP11 have not been established.

Emerging evidence has shown that ZIP transporters are associated with the development of various types of cancer. In the particular case of ZIP11, early gene association analyses using genome-wide association study (GWAS) datasets coupled with analyses of tumors for somatic change of *ZIP11* gene variants, and patient survival from data in The Cancer Genome Atlas (TCGA) showed that the variant (rs8081059) was significantly associated with increased risk of renal cell carcinoma, while four other variants (rs11871756, rs11077654, rs9913017, and rs4969054) were significantly associated with bladder cancer risk. These variants were located within predicted transcribed or enhancer regions. Moreover, out of 253 bladder cancer patients reported in TCGA, two had tumors that contained deleterious missense mutations in *ZIP11*. These data led to the identification of *ZIP11* as a contributor to bladder cancer (Wu et al., 2015). A recent study of patients with pancreatic adenocarcinoma (PAAD) showed that patients present with decreased serum Zn levels. Analysis of TCGA and the Genotype-Tissue Expression (GTEx) databases showed a correlation between high expression of *ZIP11* and poor prognosis in PAAD patients (Zhu et al., 2021). Gene expression analyses showed that *ZIP11* is upregulated in PAAD tumors compared to normal pancreatic controls (Zhu et al., 2021). KD of *ZIP11* in Capan-1 pancreatic cancer cells impaired cell proliferation associated with a decreased activation of ERK1/2 pathway (Zhu et al., 2021). A transcriptome analysis focused on colorectal cancer (CRC) and breast cancer samples showed that *ZIP11* is also upregulated in these patients (Barresi et al., 2018). Conversely, a negative correlation between *ZIP11* expression and glioma grades was described. A study involving 74 glioma tissue samples showed that low expression of *ZIP11* in gliomas correlated with grades III and IV tumors, while higher expression of the transporter correlated with grade I and II tumors (Kang et al., 2015). In this context, the data suggest that ZIP11 is a potential contributor to the development of “low grade” tumors that do not spread out of the brain, but instead grow into the normal brain tissue. However, despite this evidence, there is no information on the mechanism or detailed biological function of ZIP11 in the onset and progression of brain or other types of cancer.

In this study, we characterized the contributions of ZIP11 in maintaining cell proliferation *via* regulating Zn levels in the nuclei of HeLa cells. We reduced the expression of *ZIP11* in HeLa cells using two short hairpin RNA (shRNA) against the *SLC39A11* gene and then assessed the proliferation capabilities and Zn accumulation in whole, cytosolic, and nuclear fractions. We then treated with increasing concentrations of ZnSO_4_ and tested for metal resistance. Our data show that decreased ZIP11 expression impaired growth under normal culture conditions and increased the sensitivity of the cells due to Zn accumulation in the nuclei. RNA-seq analyses showed that the Notch pathway is downregulated in cells lacking *ZIP11.* RNA-seq and qPCR analyses revealed that the expression of cell cycle related genes was altered. For instance, we found that genes related to cell growth, such as Cyclin Dependent Kinase 20 (*CDK20*), is downregulated. On the other hand, genes implicated in the negative control of cell growth and division, such as Cyclin Dependent Kinase Inhibitor 2C (*CDKN2C*) and the Protein Phosphatase 2 Catalytic Subunit Alpha (*PPP2CA*), are induced. These analyses also showed that some DNA repair and senescence associated genes, as well as some apoptotic and genes related to epithelial mesenchymal transition (EMT), are downregulated, suggesting that the impaired growth may be due to the induction of a senescent state in the cells. Expression of exogenous wild type (WT) ZIP11 rescues the proliferation defect, restores nuclear Zn levels, and ameliorates the metal resistance phenotype observed in *ZIP11* KD HeLa cells. Interestingly, overexpression of ZIP11 in WT HeLa cells enhanced cell growth and resistance to higher levels of Zn in the media, while maintaining similar levels of the metal in cytosol and nuclei, compared to controls. Functional analyses of cancer cell migration and invasion phenotypes demonstrated that ZIP11 KD decreases the mobility and invasive capabilities of HeLa cells. As expected, ZIP11 reconstitution experiments restored these metastasis-associated properties, while overexpression of ZIP11 enhanced these phenotypes. Finally, ZIP11 KD cells have a significant decrease in mitochondrial potential and elevated β-galactosidase activity, which may also be a reflection of the dormant, or senescent, state (Chapman et al., 2019) and other metabolic deficiencies. We conclude that ZIP11 is required to maintain nuclear levels of Zn to enable proper gene expression and proliferation in HeLa cells by impairing the machinery associated with DNA damage and maintaining the cells in a senescent state. This nuclear Zn dyshomeostasis is reflected in defective metastatic properties, making ZIP11 a new potential target for further investigation using *in vivo* models and anti-cancer drug development.

## Materials and Methods

### Database Searches

We queried the publicly available database cBioPortal for Cancer Genomics, (https://www.cbioportal.org/), for SNPs within the coding region of *ZIP11* in patients with either cervical or ovarian cancer. To determine if the ZIP11 coding SNPs A234P and P243S had individual biological consequences, we queried the publicly available consensus classifier PredictSNP1 (https://loschmidt.chemi.muni.cz/predictsnp1/ (Bendl et al., 2014)). To evaluate the effects of A234P and P243S (alone or in combination) on the ZIP11 protein structure, we incorporated the SNPs into a published model of ZIP11 (AF-Q8N1S5-F1) using the PyMol Molecular Graphics System version 2.4.1 (https://pymol.org; Schrödinger, LLC). To investigate the predicted consequences that A234P and P243S might have on ZIP11s protein structure, we queried the publicly available PredictProtein algorithm (https://predictprotein.org/ (Rost et al., 2004)). To verify if the HeLa cell line was appropriate for our studies, we queried the interactive HeLa Spatial Proteome Database (http://mapofthecell.biochem.mpg.de/index.html) (Itzhak et al., 2016). To determine if wild type HeLa cells contained either the A234P or the P243S mutation within the ZIP11 gene within of its genome, we queried the Broad Institutes DEPMAP Portal (https://depmap.org/portal/cell_line/HELA_CERVIX?tab=mutation). The https://www.ncbi.nlm.nih.gov/gene?Db=gene&Cmd=DetailsSearch&Term=201266 website was used to identify the number of ZIP11 isoforms that may be present in cells.

### Cell Culture

HeLa and HEK293T cells were obtained from American Type Culture Collection (ATCC, Manassas, VA, United States) and cultured in DMEM media (Sigma-Aldrich, St Louis, MO, United States) supplemented with 10% fetal bovine serum (FBS) and 1% antibiotics (penicillin G/Streptomycin, Gibco, Waltham, MA, United States) in a humidified atmosphere containing 5% CO_2_ at 37°C.

### Plasmids and Lentivirus Production

Mission plasmids encoding for two different shRNA against human *ZIP11* and the control scrambled (Scr) construct with a puromycin resistance cassette were obtained from Sigma (Supplementary Table S1). The mammalian gene expression lentiviral vector pLV[Exp]-EGFP/Neo-EF1A encoding hSLC39A11 or empty vectors with a neomycin resistance cassette were purchased from Vector Builder. Plasmids were isolated with the ZymoPURE™ II maxiprep plasmid system (Zymo Research, Irvine, CA, United States) following the manufacturer’s instructions. shRNA (15 µg) and the packing vectors pLP1 (15 µg), pLP2 (6 µg), pSVGV (3 µg) were transfected using lipofectamine 2000 (Thermo Fisher, Waltham, MA, United States) into HEK293T cells for lentiviral production. After 24 and 48 h, the supernatant containing viral particles were collected and filtered using a 0.22 µm syringe filter (Millipore Sigma, Burlington, MA, United States). HeLa cells were transduced with lentivirus in the presence of 8 mg/ml polybrene and selected with 4 μg/ml puromycin (Invitrogen, Waltham, MA, United States) or 2 mg/ml geneticin. After selection, the cells were maintained with 1 μg/ml of puromycin or 200 μg/ml of geneticin as needed.

### Antibodies

The rabbit anti-ZIP11 (PA5-20679), antibody was from Thermo Fisher. The mouse anti-lamin A/C (SC376248) and anti-tubulin (TU-02; SC8035) were from Santa Cruz Biotechnologies (Dallas, TX, United States). The rabbit anti-GAPDH (A19056) and anti-MTF1 (custom made against the residues 520–630 from the human protein) were from Abclonal Technologies (Woburn, MA, United States). The rabbit anti-Caspase-3 antibody was from Cell Signaling Technologies (9662). The mouse anti-Golgin-97 (A21270) and the secondary HRP-conjugated anti-mouse and anti-rabbit antibodies were from Invitrogen (31,430 and 31,460, respectively). The fluorescent goat anti-rabbit Alexa-488 secondary antibody was from Thermo Fisher (A-11008).

### Western Blot Analyses

Protein samples from HeLa cells (WT, Scr control, *ZIP11*-KD, and cells transduced with the empty or ZIP11-containing pLV[Exp]-EGFP/Neo-EF1A vectors) were solubilized with RIPA buffer (10 mM piperazine-N,N-bis(2-ethanesulfonic acid), pH 7.4, 150 mM NaCl, 2 mM ethylenediamine-tetraacetic acid (EDTA), 1% Triton X-100, 0.5% sodium deoxycholate and 10% glycerol) supplemented with protease inhibitor cocktail (Thermo Fisher). Protein content was quantified by Bradford assay (Bradford, 1976). Samples (20 µg) were separated by SDS-PAGE and electrotransferred to PVDF membranes (Millipore Sigma). The proteins of interest were detected using the primary antibodies anti-ZIP11 and anti-GAPDH as a loading control. The membranes were then incubated with species-specific secondary antibodies coupled to horseradish peroxidase. Chemiluminescent detection was performed using high sensitivity Tanon reagents (Abclonal Technologies).

### Confocal Microscopy

Monolayers of HeLa cells were fixed overnight in 10% formalin-PBS at 4°C. Samples were washed with PBS, permeabilized with 0.2% Triton X-100 in PBS for 15 min, incubated for 1 h at RT in blocking solution (PBS, 0.2% Triton X-100, 3% FBS), and incubated overnight with anti-ZIP11 and anti-Golgin 97 antibodies in blocking buffer at 4°C. The next day, the cells were incubated for 3 h with fluorescent goat anti-rabbit Alexa-594 and anti-mouse Alexa-633 secondary antibodies in blocking solution for at RT and 30 min with DAPI. Microscopy and image processing were performed using a Leica SP8 Confocal Microscope and the Leica Application Suite X (Leica Microsystems Inc., Buffalo Grove, IL, United States).

### Cell Proliferation Assays

HeLa and HeK293T cells were seeded at 1 × 10^4^ cells/cm^2^ and samples were collected 24, 48, 72, and 96 h after plating. Increasing concentrations of ZnSO_4_ (0–200 µM) were added to the cell cultures as indicated in the figures and figure legends. The cells were trypsinized, washed three times with PBS, and counted using a Cellometer Spectrum (Nexcelcom Biosciences, Lawrence, MA, United States). To determine cell viability, HeLa cells were collected at 72 h after plating and stained with 0.4% Trypan Blue (Sigma) diluted in PBS for 5 min at RT. Cell number and viability were determined using the Cellometer Spectrum, and data were analyzed with FCS Express 7 software (*De Novo* Software).

### Metal Content Analysis

Three independent biological replicates of HeLa cells stably expressing the shRNA or the pLV[Exp]-EGFP/Neo-EF1A encoding hSLC39A11 or empty vectors were seeded at 1 × 10^4^ cells/cm^2^ and allowed to proliferate for 48 h. Then the cells were rinsed three times with ice-cold PBS without Ca^2+^ and Mg^2+^ (Gibco). Subcellular fractionation was performed following the Rapid, Efficient, and Practical nuclear and cytoplasmic separation method (Suzuki et al., 2010; Gordon et al., 2019a; Tavera-Montañez et al., 2019). Briefly, cells were scraped and transferred to a 1.5-ml microcentrifuge tube. Cells were centrifuged for 10 s at 13,000 × *g* and the supernatant was discarded. The samples were resuspended in 400 ml of ice-cold PBS containing 0.1% NP40 (Sigma-Aldrich) and 50 µl of the cell suspension were collected as the whole cell fraction. The remaining 350 µl were used to obtain nuclear and cytosolic fractions by disrupting the cells by pipetting using a 1-ml pipette tip. Cell suspension was centrifuged for another 10 s and the supernatant was collected as the cytosolic fraction. The nuclear pellet was washed twice in 1 ml of ice-cold PBS containing 0.1% NP40 and centrifuged for additional 10 s. The supernatant was removed, and the pellet was resuspended in 100 µl of PBS. Nuclear integrity was verified by light microscopy. All samples were sonicated at medium intensity for 5 min in 30 s on 30 s off cycles. Protein was quantified by the Bradford method (Bradford, 1976). Purity of cytosolic and nuclear fractions used to determine metal levels was verified by western blot using an anti-Tubulin and anti-Lamin A/C antibodies.

The comparative analysis of ultra-trace (<1 ppm) Zn concentrations from each sample was determined using a method adapted from previously described protocols (Paskavitz et al., 2018; Gordon et al., 2019a; Tavera-Montañez et al., 2019). Here, Zn measurements were carried out using a PerkinElmer AAnalyst 800 atomic absorption spectrometer (AAS) with a zinc hollow cathode lamp as the radiation source. The AAS was equipped with a graphite furnace (GF-AAS) with UltraClean THGA^®^ graphite tubes (PerkinElmer, Waltham, MA, United States). This technique allowed accurate ultra-trace zinc analysis in low volume samples, where dilution was limited by the low initial concentration of Zn in the samples (Paskavitz et al., 2018; Gordon et al., 2019a). In a typical analysis, a known mass of the sample was digested in concentrated nitric acid using single-stage digestion (Paskavitz et al., 2018; Gordon et al., 2019a; Tavera-Montañez et al., 2019). The resulting solution was analyzed for Zn *via* AAS with measurements carried out at least in triplicates. Contamination was avoided by using analytical grade reagents and 18 MΩ purified water. All analytical glassware was acid washed overnight in 10% (v/v) hydrochloric acid and rinsed with 18 MΩ purified water before use (Paskavitz et al., 2018; Gordon et al., 2019a; Tavera-Montañez et al., 2019; Kim et al., 2020). Zn standard solutions were prepared from 1000 mg/L (Sigma-Aldrich) to determine the limits of detection and obtain a calibration curve for the method. The limit of detection for Zn, calculated as three times the standard deviation of the intercept (3σ), was 0.05 ppb, with a limit of linearity at 2.5 ppb. Zn content on each sample was normalized to the initial protein content in each sample.

### RNA-Seq and Data Analysis

Total RNA from HeLa cells transduced with Scr or one of two shRNA against *ZIP11* was isolated using TRIzol and frozen at −80°C until analysis. Independent replicates for each sample were evaluated for quality and concentration at the Molecular Biology Core Lab at the University of Massachusetts Chan Medical School. Quality Control-approved samples were submitted to BGI Genomics for library preparation and sequencing. Libraries were sequenced using the BGISEQ-500 platform and reads were filtered to remove adaptor-polluted, low quality and high content of unknown base reads. About 99% of the raw reads were identified as clean reads (∼65 M). The resulting reads were mapped onto the reference human genome (hg38) using HISAT (Kim et al., 2015). Transcripts were reconstructed using StringTie (Pertea et al., 2015), and novel transcripts were identified using Cufflinks (Trapnell et al., 2010) and combined and mapped to the hg38 reference transcriptome using Bowtie2 (Langmead and Salzberg, 2012). Gene expression levels were calculated using RSEM (Li and Dewey, 2011). DEseq2 (Love et al., 2014) and PoissonDis (Audic and Claverie, 1997) algorithms were used to identify differentially expressed genes (DEG). Gene Ontology (GO) analysis was performed on DEGs to cluster genes into function-based categories.

### RT-qPCR Gene Expression Analysis

RNA was purified from three independent biological replicates of proliferating HeLa cells (Scr control and KDs) with TRIzol (Invitrogen) following the manufacturer’s instructions. cDNA synthesis was performed with 500 ng of RNA as template, random primers, and SuperScript III reverse transcriptase (Invitrogen) following the manufacturer’s protocol. Quantitative RT-PCR was performed with Fast SYBR green master mix on the ABI StepOne Plus Sequence Detection System (Applied Biosystems) using the primers listed in Supplementary Table S2, and the delta threshold cycle value (ΔC_T_) (Livak and Schmittgen, 2001) was calculated for each gene and represented the difference between the C_T_ value of the gene of interest and that of the control gene, *GAPDH.*

### Wound Healing Assay

Cells were grown until confluence on 24 well plates in DMEM supplemented with 10% FBS and antibiotics. Cells were starved for 24 h in DMEM without FBS and cell proliferation was inhibited by treating the cells with Cytosine β-D-Arabinofuranoside (AraC) for 2 h. The monolayers were then scratch-wounded using a sterile 200 μl pipette tip and suspended cells were washed away with PBS twice. The progress of cell migration into the wound was monitored every 24 h until wound closure using the ×10 objective of an Echo Rebel Microscope as previously described (Lacombe et al., 2021). The bottom of the plate was marked for reference, and the same field of the monolayers was photographed immediately after performing the wound (time = 0 h) and at different time points after performing the scratch, as indicated in the figures. Area migrated by the cells was quantified using FIJI software, version 1.44p (Schindelin et al., 2012).

### Matrigel Invasion Assay

Matrigel invasion assay was performed following the Transwell chamber method as described (Olea-Flores et al., 2019). Briefly, BioCoat^®^ Matrigel^®^ Invasion Chambers with 8.0 µm PET membrane placed in 6-well Plates were used to seed cells that were previously treated for 2 h with 10 µM AraC to inhibit cell proliferation. The cells were plated at 1.25 × 10^5^ cells/ml in 2 ml of serum-free medium on the top chamber, as recommended by the manufacturer. The lower chamber of the Transwell contained 2.5 ml of advanced DMEM supplemented with 10% FCS. Cells were incubated for 24 h at 37°C in a 5% CO_2_ atmosphere. Following incubation, cells and Matrigel on the upper surface of the Transwell membrane were gently removed with cotton swabs. Invading cells on the lower surface of the membrane were washed and fixed with methanol for 5 min and stained with 0.1% crystal violet diluted in PBS. Images from 10 fields of three independent biological replicates were taken and used for cell quantification using FIJI software, version 1.44p (Schindelin et al., 2012). The invasion index was calculated as the ratio between number of cells of *ZIP11* KD cells, KDs reconstituted with EV or *ZIP11*, or WT overexpressing the EV or *ZIP11* and the number of WT control cells.

### Cell Cycle Analyses

HeLa cells (1 × 10^6^ cells) were arrested in mitosis with 50 ng/ml nocodazole (Kaida et al., 2011) for 16 h and released by washing with PBS and cultured with medium with 10% FBS for an additional 24 h. Timepoints were collected as indicated in the figure legend. Cell cycle analysis was performed using a standard propidium iodide (PI)-based cell cycle assay. Briefly, cells were trypsinized, washed three times with PBS, and fixed by slowly adding 200 μl of ice-cold 70% ethanol and incubated overnight at 4°C. Cells were washed with PBS, and the pellet was resuspended in 50 μl PBS containing 100 μg/ml RNAse A and 0.1% Triton X-100 and incubated at 37°C for 30 min. Finally, the cells were incubated with 40 μg/ml PI staining solution at 37°C for 40 min and analyzed in a Cellometer Spectrum instrument. Data were analyzed with FCS Express 7 software.

### Senescence Assay

We used CellEvent^™^ Senescence Green Flow Cytometry Assay Kit following manufacturer’s instructions (Thermo Fisher). Briefly, HeLa cells (WT, Scr control, *ZIP11* KD, and cells transduced with the empty or *ZIP11*-containing pLV[Exp]-EGFP/Neo-EF1A vectors) were seeded at 1 × 10^6^ cells/cm^2^ and maintained on DMEM media supplemented with 10% FBS for 48 h. We treated wild type HeLa cells with 5 mM Palbociclib (Sigma-Aldrich) as a positive control for senescence, as suggested by the manufacturer. HeLa cells were trypsinized, washed and resuspended in PBS and fixed in 4% paraformaldehyde for 10 min at room temperature. The cells suspension was stained with the CellEvent^™^ Senescence Green Probe (1/500) in CellEvent^™^ Senescence Buffer for 90 min in a 37°C incubator with no CO_2_. Cells were washed in PBS containing 1% BSA, and finally resuspended in PBS. Fluorescence intensity of β-gal was measured by Spectrum Cellometer (Nexcelom Biosciences) by setting the filter excitation at 530/30 nm filter. Data was analyzed with FCS Express 7 (*De Novo* Software).

### Mitochondrial Membrane Potential

Changes in mitochondrial membrane potential produced by *ZIP11* KD in HeLa cells were determined with the tetramethylrhodamine ethyl ester (TMRE)-Mitochondrial Membrane Potential Assay Kit (Abcam, Cambridge, MA, United States) following the manufacturer’s protocol. Briefly, proliferating cells were supplemented with 200 nM TMRE and incubated in the dark for 10 min at 37°C. The cells were then trypsinized and washed three times with PBS. Fluorescence intensity of TMRE was measured by Spectrum Cellometer (Nexcelom Biosciences, Lawrence, MA, United States) by setting the filter excitation at 502 nm and emission at 595 nm, as previously reported (Angireddy et al., 2020; Chowdhury et al., 2020; Lacombe et al., 2021). Data was analyzed with FCS Express 7 (*De Novo* Software).

### Statistical Analysis

Statistical analyses were performed using Kaleidagraph (Version 4.1). Statistical significance was determined using *t*-test where *p* < 0.05 was considered to be statistically significant.

## Results

### ZIP11 Plays a Role in the Progression of Cervical and Ovarian Cancer

A closer look to the TCGA database showed that of 1321 cases reported in the TCGA database for *ZIP11* (SLC39A11) mutations, 61% have been found in females and 39% in males. The higher incidence of mutations for *ZIP11* gene occurs in patients presenting uterine corpus endometrial carcinoma (Supplementary Figure S1A). In terms of loss of function or decrease expression of the *ZIP11* gene esophageal cancer patients represent the most affected population (8% of the patients), while for gain of function or increased expression of *ZIP11*, impacts primarily ovarian cysteous adenocarcinoma (almost 30% of the patients), followed by breast invasive carcinoma (approximately 22% of the individual; Supplementary Figure S1B). Between 10 and 15% of the patients presenting lung squamous cell carcinoma, bladder urothelial carcinoma, esophageal carcinoma, uterine corpus endometrial and cervical squamous cell and endocervical carcinomas are also among the groups presenting increased expression of *ZIP11* (Supplementary Figure S1C).

**FIGURE 1:**
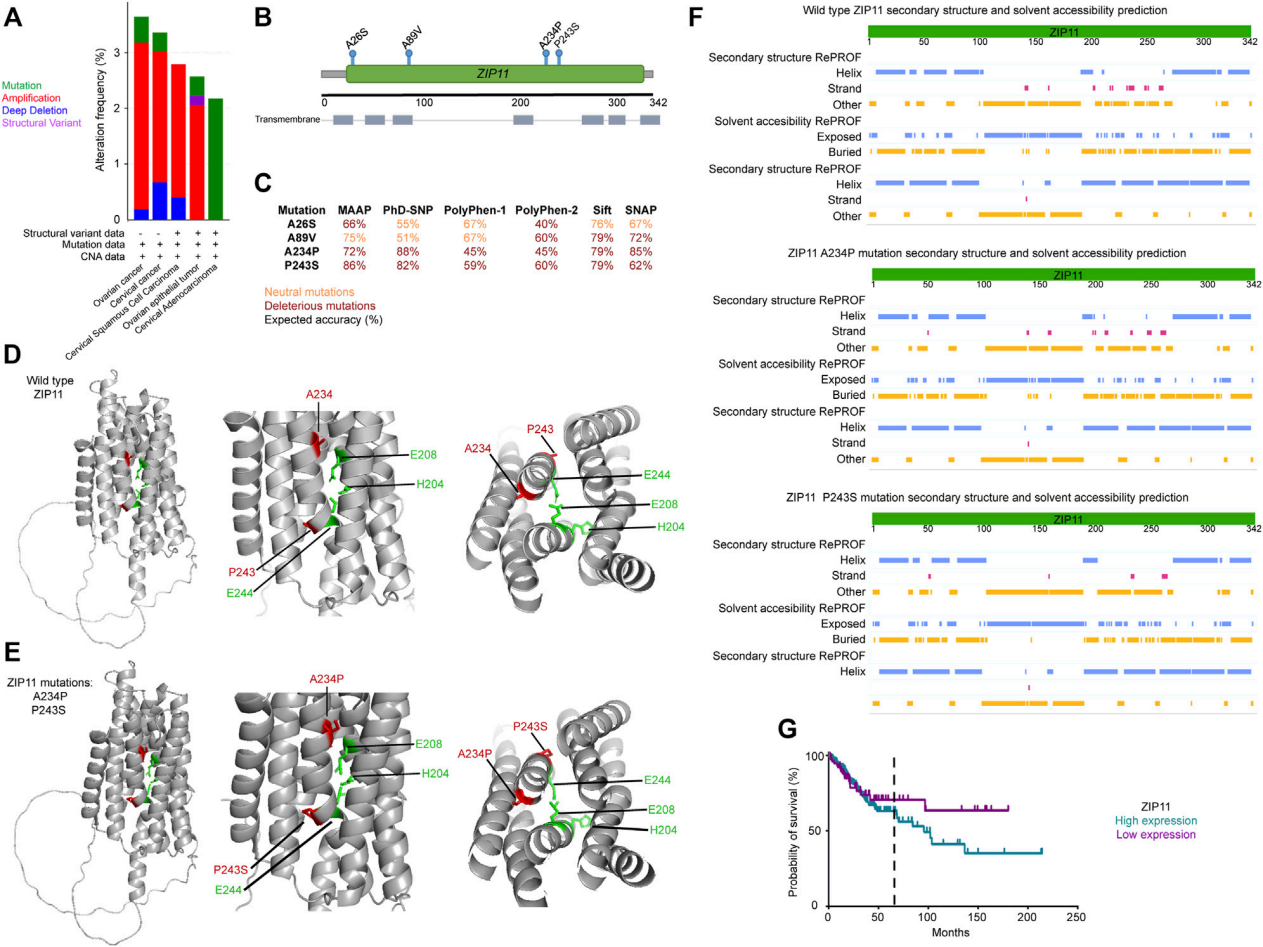
ZIP11 role in the progression of cervical and ovarian cancer. **(A)** Analysis of 2344 samples from 2330 patients found in seven publicly available cervical and ovarian cancer studies (https://www.cbioportal.org/) confirmed that ZIP11 had an alteration frequency of 2–3%, within the genome. **(B)** Schematic of ZIP11 and SNP locations (green) within the ZIP11 protein and transmembrane domains (gray). **(C)** PredictSNP analysis of *ZIP11* SNPs in cervical and ovarian cancer (red = deleterious; orange = neutral mutations). Representative model of wild type ZIP11 **(D)** and ZIP11 containing two deleterious SNPs (A234P and P243S, **(E)** within the substrate binging region. **(F)** *In silico* modeling with PredictProtein calculated that ZIP11 may have increased substrate accessibility when the two deleterious SNPs (A234P and P243S) are introduced. **(G)** Kaplan-Meier survival curve using a cervical cancer data set containing 291 patients. Five-year survival indicated by black, vertical, dashed line. The statistical information for the comparison of survival curves can be found in Supplementary Table S3.

Patients of cervical and ovarian cancers represent the groups with larger numbers in *ZIP11* mutations, however mutations on this gene are not considered to be a prognostic marker of the disease (Figure 1 and Supplementary Figure S1 and Supplementary Table S3). Therefore, we looked closely into genotypes and phenotypes observed in these populations. Analysis of 2344 samples from 2330 patients found in seven publicly available cervical and ovarian cancer studies (https://www.cbioportal.org/) confirmed that *ZIP11* had an alteration frequency of 2–3%, within the genome, with the majority of the genes being amplified or mutated rather than deleted (Figure 1A). Further structural analysis of these datasets revealed two unique missense mutations within the *ZIP11* coding region, A26S and A234P, while another two mutations, P243S and A89V, correlated with the two known coding SNPs rs763797008 and rs202154945 (Figure 1B). Although the effect of an individual SNP is generally minor, some variants do affect gene expression or the function of the translated proteins (Risch and Merikangas, 1996; Collins et al., 1997). Therefore, the effect of combinations of functionally relevant SNPs may synergistically contribute to increased disease progression. Compared to individual SNPs, multiple SNPs can be either more or less deleterious. Multiple SNPs on the same gene have been found to contribute or be linked to various genetic diseases (Rafi et al., 2003; Kamphans et al., 2013). To determine if these SNPs had biological consequences, we used PredictSNP (Bendl et al., 2014), a publicly available consensus classifier for disease related amino acid mutations. Results showed that both A234P and P243S had deleterious biological consequences (Figure 1C). The limitation of the PredictSNP model is that it can only predict the consequence of one SNP, not multiple. We knew that some of the patients in these data sets were positive for both A234P and P243S mutations. Therefore, using PyMol Molecular Graphics System, we used the predicted structure of ZIP11 (PDB AF-Q8N1S5-F1) to construct a model that contained the two deleterious SNPs and compared this to the wild type structure. The model indicated SNP induced structural variations within the substrate binding region that possibly could affect the function of the protein (Figures 1D,E). To show exactly where the SNPs affected ZIP11 structure and what could be the predicted consequences; we used the publicly available PredictProtein algorithm. PredictProtein searches public sequence databases, creates alignments, and predicts aspects of protein structure and function (Rost et al., 2004). These *in silico* analyses suggested that substitutions in A234P and P243S may result in an increase in substrate accessibility (Figure 1F). Since these residues are facing away from the transmembrane metal binding site, these mutations may potentially affect the interactions between surrounding transmembrane helices. However, further biochemical characterization is needed to clarify this point. Finally, using the Human Protein Atlas (https://www.proteinatlas.org/) we constructed a Kaplan-Meier survival curve using a cervical cancer data set containing 291 patients. The survival curve demonstrated that patients with high *ZIP11* RNA expression within their cervical cancer tumors had a 63% chance of surviving over 5 years, while those with low *ZIP11* RNA had a 72% survival rate (Figure 1G and Supplementary Table S3). This trend in survival suggests that ZIP11 expression may correlate with the progression of cervical cancer, although the information provided by the database does not specify whether these patients harbor the indicated SNPs.

ZIP11 has been proposed to be a transporter that mobilizes Zn from the nucleus and Golgi into the cytosol (Kambe et al., 2015). However, overexpression studies in early characterization of RAW264.7 cells, which are monocyte/macrophage-like cells suggested a potential role mobilizing extracellular Zn into the cytosol (Yu et al., 2013). To verify if the HeLa cell line was an appropriate model for our studies of ZIP11 in the context of nuclear transport, we used the interactive HeLa Spatial Proteome Database (http://mapofthecell.biochem.mpg.de/index.html) to understand the cellular localization of ZIP11. The HeLa Spatial Proteome Database provides subcellular localization information for 8,700 proteins from HeLa cells. It indicates how a queried protein is distributed over the nucleus, cytosol, and organelles of the HeLa cells using a cross-validation of a 1,000-member organelle marker set resulting in a median prediction accuracy of >94% (Itzhak et al., 2016). Principal components analysis of six Dynamic Organellar Maps of ZIP11 generated by the HeLa Spatial Proteome interactive (Supplementary Figures S2A–F) database revealed that ZIP11 is present in both the nucleus and some organelle compartments of HeLa cells in roughly equal numbers and is also present in the cytosol (*p* = 0.0269 when comparing nucleus to cytosol; (Supplementary Figure S2G). Finally, using the DEPMAP portal, we confirmed that the wild type HeLa cell line does not harbor either the A234P or the P243S mutation in the ZIP11 gene within its genome (https://depmap.org/portal/cell_line/HELA_CERVIX?tab=mutation). Taken together, the HeLa cell line was determined to be an appropriate model for our studies of ZIP11 in the context of nuclear transport.

### *ZIP11* Expression Is Required for Proliferation in HeLa Cells

**FIGURE 2:**
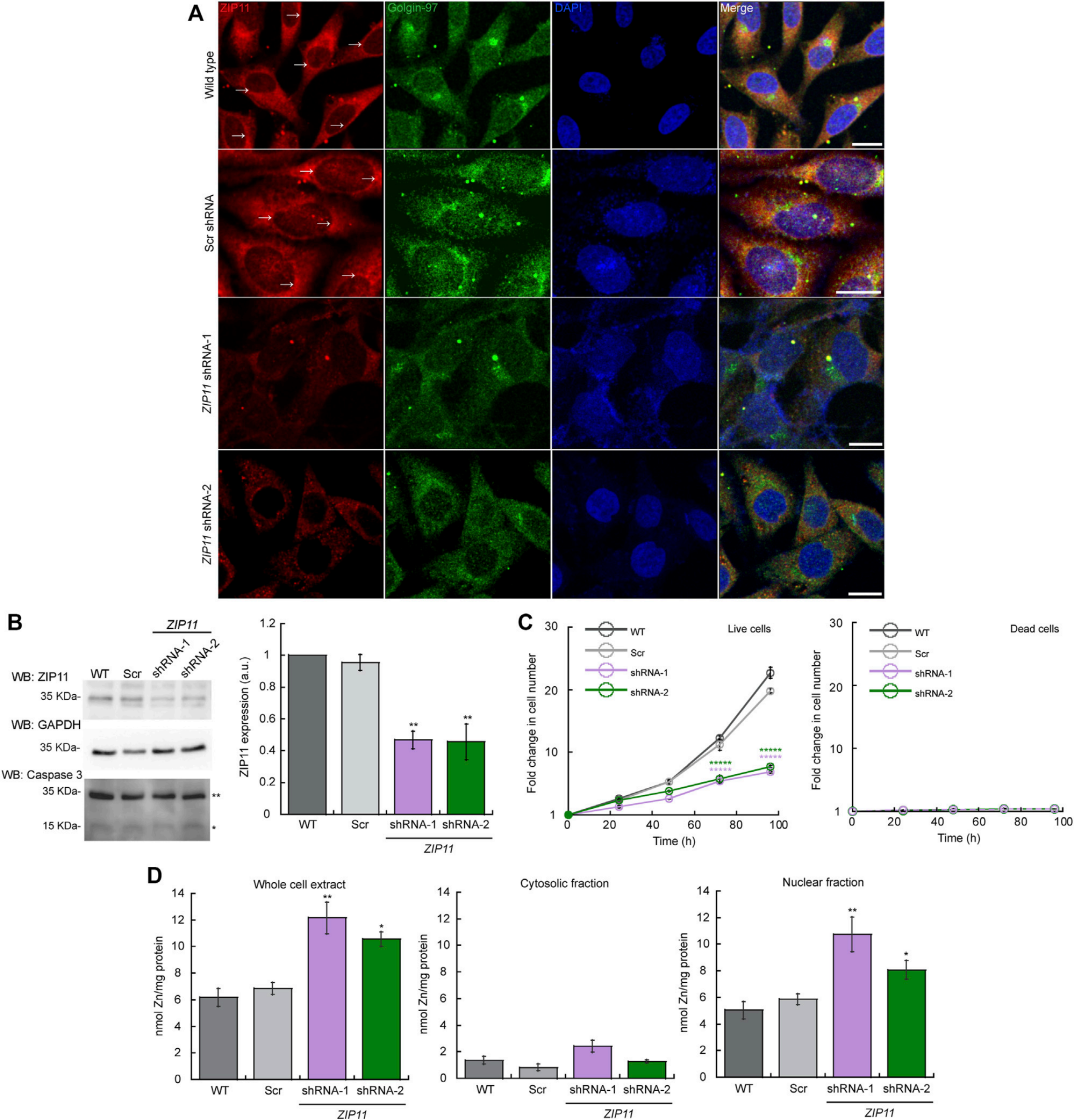
ZIP11 is required for proliferation of HeLa cells and regulates nuclear levels of Zn. **(A)** Representative confocal images showing a perinuclear and cytosolic punctuated pattern of expression of ZIP11 (red) in wild type and scramble (Scr) shRNA transduced HeLa cells. Confocal images of HeLa cells transduced with *ZIP11* shRNA-1 and shRNA-2 show a decrease in the staining around the nucleus and vesicles. shRNA1 and shRNA2 targets the CDS and UTR regions of *ZIP11*, respectively. The anti-Golgi-97 antibody (green) was used as a marker of Golgi apparatus and nuclei was stained with DAPI (blue). Bar = 10 µm. **(B)** Representative immunoblot (left) and quantification (right) of *ZIP11* levels of the 35 KDa (isoform 1) in HeLa proliferating cells for 72 h. *See* Supplementary Figure S3 for the data of the rest of isoforms. Immunoblots against GAPDH were used as loading controls. Samples were compared to the corresponding wild type sample. An anti-caspase 3 antibody was used to detect cell death in HeLa cells proliferating for 72 h; single asterisk (*) indicate cleaved caspase 3 and double asterisks (**) indicate the full inactive pro-caspase 3 form. **(C)** Cell counting assay of proliferating WT, and cells transduced with scrambled shRNA (shRNA Scr), or *ZIP11* shRNAs. Data represent the fold change in number of live and dead cells as determined by Trypan blue assay. **(D)** Zn levels in proliferating HeLa cells. Wild type and cells stably expressing Scr, shRNA-1 or shRNA-2 against *ZIP11* were allowed to grow for 48 h and subcellular fractions were obtained using the REAP protocol. Whole cell (left) cytosolic (middle) and nuclear (right) Zn content determined by AAS (Suzuki et al., 2010; Gordon et al., 2019a; Tavera-Montañez et al., 2019). For all samples, data are the mean ± SE of three independent biological replicates. *p < 0.05; **p < 0.01; *****p < 0.00001.

Considering that cervical cancer patients are amongst the higher incident population of individuals with mutations in *ZIP11*, we chose HeLa cervical carcinoma cells as a model to investigate the contributions of this transporter to the cancer phenotype. ZIP11 has been proposed to localize in the nuclei and Golgi, and confocal microscopy analyses of WT cells shows that ZIP11 is primarily located in the perinuclear/nuclear region of the cells and partially colocalized with the Golgi marker Golgin-97 vesicles (Figure 2A). Furthermore, we performed confocal microscopy analyses of ZIP11 and the endoplasmic reticulum (ER) marker calnexin and detected minimal colocalization between these proteins, suggesting no strong association of ZIP11 with the ER on HeLa cells (Supplementary Figure S3).

To test the biological role of ZIP11 in the proliferation of HeLa cells, we reduced the expression of the transporter using two different shRNA clones, one which targeted the coding sequence (sh-1) and the other the UTR region (sh-2). Confocal microscopy (Figure 2A), and western blot and densitometric analyses of three independent biological replicates (Figure 2B) showed the decreased expression of *ZIP11* following this strategy and confirms the specificity of the ZIP11 antibody. According to the https://www.ncbi.nlm.nih.gov/gene?Db=gene&Cmd=DetailsSearch&Term=201266 website, there are 16 known isoforms of the ZIP11 protein expressed in human cells that range from 20 to 36 KDa (Supplementary Figure S3). Analyses of the complete membrane showed that the selected antibody likely recognize some of these variants of ZIP11 (Supplementary Figure S3). The specific roles of these isoforms are beyond the scope of this work; however we can detect a reduction on their expression with the two shRNAs used in this study. Two higher MW bands are present on the gel. We believe these are non-specific targets in western blot analyses, as quantification of the individual bands/clusters of bands indicates these species are not reduced by the ZIP11 shRNAs whereas the ZIP11 isoforms are reduced by the shRNAs. We focused on the 35 KDa isoform (X1) for our study as it is one of the most common forms expressed in human cells.

Upon reduction of the expression of ZIP11, we detected a significant decrease in the proliferation rate of KD cells (Figure 2C left panel). Trypan blue analyses determined that there is no increase in cell death in *ZIP11* KD cells during the experiment (Figure 2C right panel), which was verified by western blot analyses against caspase 3 of proliferating HeLa cells at a representative time point of 72 h (Figure 2B). We also tested the effect of *ZIP11* KD in the non-carcinogenic cell line HEK273T, isolated from human embryonic kidneys. We observed that upon KD of the transporter there are no significant changes in the proliferation of these cells (Supplementary Figure S2). The contrasting results of the effect of *ZIP11* KD on the proliferation rate observed between HeLa and HEK cells suggest that the biological role of the transporter may be dependent on the cellular context and may be a distinctive feature across cell types and tissues.

Therefore, we focused our studies in HeLa cells, as we observed the most dramatic phenotype associated to *ZIP11* KD in this cell line. Considering the location of the transporter, we evaluated the effect of ZIP11 KD on metal accumulation in whole cell extract, cytosolic and nuclear fractions of HeLa cells (Figure 2D left, middle and right panels, respectively). ZIP11 has been implicated in nuclear transport of Zn because its expression is dependent on Zn levels; however, *ZIP11* does not exhibit drastic changes upon cellular exposure to this metal, as other metalloprotective genes (Martin et al., 2013; Yu et al., 2013). Therefore, to understand how partial loss of ZIP11 affects accumulation of Zn, we used AAS to measure total metal levels in proliferating HeLa cells (Figure 2D). WT and cells transfected with scrambled (Scr) shRNA showed similar content of Zn in whole cell extracts, with Zn mainly located in the nucleus. Importantly, a significant increase in whole cell levels of Zn was observed in the two *ZIP11* KD cell lines with the metal accumulated primarily in the nuclei (Figure 2D), suggesting that reduced expression of this transporter impairs the mobilization of the ion from the nucleus to the cytosol. Supplementary Figure S6 shows a representative western blot of the purity of the cytosolic and nuclear fractions used in the metal content analyses. Tubulin was used as a marker of cytosol and Lamin A/C was used as marker of the nuclear fractions.

**FIGURE 3:**
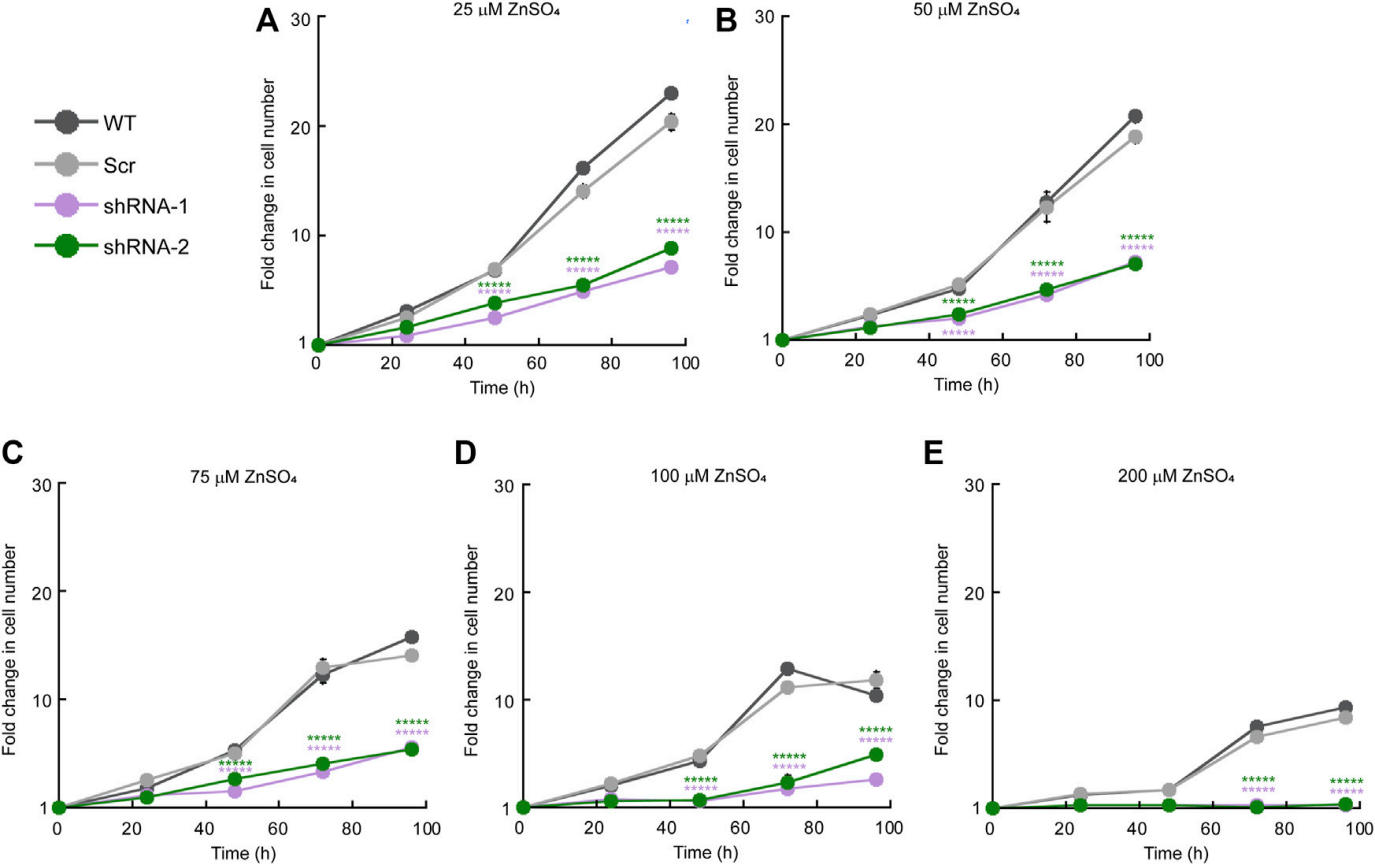
*ZIP11* knockdown results in elevated sensitivity to Zn in HeLa cells. Cell counting assay of proliferating WT, and cells transduced with scrambled shRNA (shRNA Scr), or *ZIP11* shRNAs cultured for 96 h with increasing concentrations of ZnSO_4_. **(A)** 25 µM. **(B)** 50 µM. **(C)** 75 µM. **(D)** 100 µM. **(E)** 200 µM. Cells were seeded at 1 × 10^4^ cells/cm^2^ and the growth for each clone and timepoint was normalized to the seeding density. The data represents the mean ± SE for three independent experiments. *****p < 0.00001.

We then analyzed the effect of Zn stress on the growth of HeLa cells KD for ZIP11 (Figure 3). To this end, cells were cultured in the presence of increasing concentrations of ZnSO_4_, and counting assays were performed over 5 days. We found that the KD cells had a decrease in proliferation at 75 µM ZnSO_4_ (Figure 3C) compared to non-treated KD and control cells, which were sensitive to higher concentrations of the metal (200 μM; Figures 3D,E). These data suggest that ZIP11 may be a regulator of proliferation in HeLa cells by mediating nuclear Zn homeostasis, by potentially mediating appropriate gene expression. However, we cannot overrule alternative mechanisms that lead to a different pathway where *ZIP11* KD also influences the levels of Zn in Golgi and potentially other subcellular organelles which have not been isolated yet, to produce this deleterious effect.

### Effect of *ZIP11* KD in HeLa Cells Transcriptome

**FIGURE 4:**
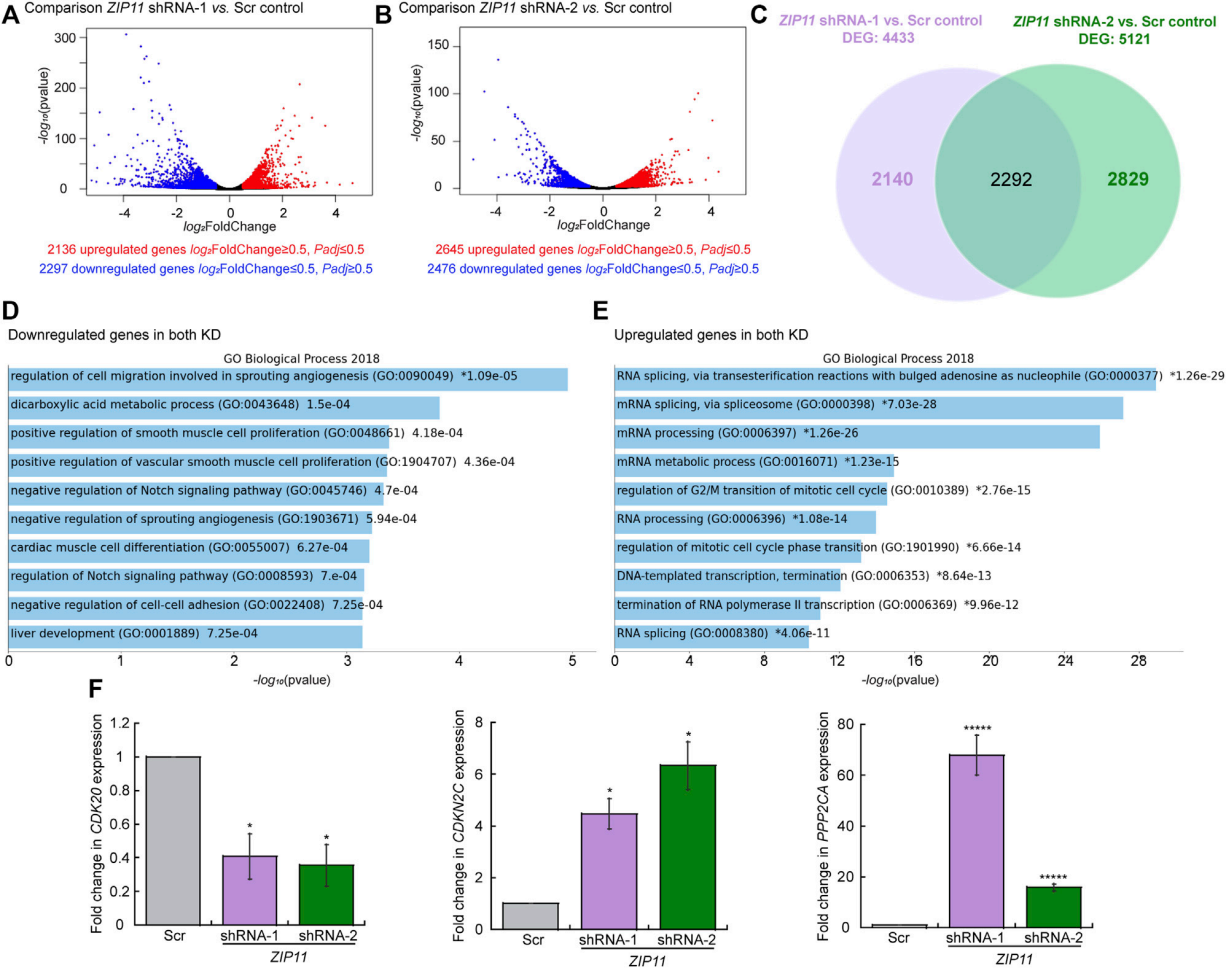
Changes in gene expression dependent on *ZIP11* knockdown in HeLa cells. Volcano plots displaying differentially expressed genes between Scr control and *ZIP11* KD with shRNA1 **(A)** or*shRNA2* **(B)** in HeLa cells. The *y*-axis corresponds to the mean log10 expression levels (*p* values). The red and blue dots represent the up- and down-regulated transcripts in *ZIP11* KD (false-discovery rate [FDR] of <0.05), respectively. The gray dots represent the expression levels of transcripts that did not reach statistical significance (FDR of >0.05). **(C)** Venn diagram showing the overlapping DEG between the two shRNAs used to KD *ZIP11*. GO term analysis of down-regulated **(D)** or up-regulate **(E)** genes consistent in both KD of *ZIP11* in HeLa cells. Cut-off was set at 2.0 of the −log(adjusted *p* value). *See* Supplementary Table S4 for the complete list of genes and individual GO terms detected for each shRNA using Panther. **(F)** Steady state mRNA levels determined by qRT-PCR of representative downregulated (*CDK20*) and up-regulated (*CDKN2C* and *PPP2CA*) genes selected from the RNA-seq analyses which are associated to cell cycle progression and apoptosis. Data are the mean ± SE for three independent experiments. *p < 0.05, *****p < 0.00001.

We performed RNA-seq to investigate global changes in gene expression in proliferating HeLa cells transduced with either the Scr or the two different *ZIP11* shRNAs. The sequenced libraries from the samples had approximately 92M total reads, where the average mapping ratio with the gene is 85.28%. The unique matched reads are shown in Supplementary Table S4. Reads were mapped to the human genome (GRCh38/hg38) and gene expression levels were determined. Differentially expressed genes that were significant in both replicates for each shRNA were considered for analysis (log_2_FoldChange>1 and <−1). Replicate samples for scramble and *ZIP11* shRNA resulted in Pearson coefficients of >0.94 for each comparison of replicates (Supplementary Table S4). Each *ZIP11* KD affected the expression of a similar number of genes, however, there are noticeable differences (Figures 4A–C). shRNA-1 affected a total of 4433 genes, of which 2136 were upregulated and 2297 were downregulated (Figure 4A), and shRNA-2 affected 5121 genes in total, with 2645 genes upregulated and 2476 downregulated (Figure 4B). Both shRNAs shared 2292 differentially expressed genes (DEG) compared to gene expression in the control cells (Supplementary Table S4 and Figure 4C). To identify function-based categories, we performed gene ontology (GO) analysis on DEG that were significant in both replicates for both shRNAs. The complete results are listed in Supplementary Table S4. The top 10 significant categories of down-regulated and up-regulated genes shared by both KD cells are shown in Figures 4D,E. The top ranked categories of down-regulated genes included regulation of cell migration involved in angiogenesis, metabolism of dicarboxylic acid, regulation of smooth muscle proliferation, and Notch signaling pathway (Figure 4D and Supplementary Table S4). RNA processing and metabolism were the most remarkable up-regulated categories followed by regulation of cell cycle, but genes involved in the regulation of DNA transcription and termination were also upregulated (Figure 4E and Supplementary Table S4). A close analysis of genes related to cell growth, DNA repair, senescence, apoptosis, and EMT suggested important changes that may explain the impaired proliferation phenotype and different shapes observed in both KD strains. Some of these genes were validated by qPCR analyses. For instance, the expression of the cell cycle regulatory gene *CDK20* is decreased (Figure 4F and Supplementary Table S4), and genes related to EMT (*LOXL3* and *FUZ*; Supplementary Table S4) are downregulated as well. Interestingly, we found changes in the expression of senescence associated genes, however, these behaved slightly different in both shRNA KD strains. In addition, genes implicated in the negative control of cell growth and division, such as *CDKN2C* and *PPP2CA*, were upregulated (Figure 4F). Genes implicated in senescence (*CDKNA* (*p21*) (Pospelova et al., 2009; Noren Hooten and Evans, 2017)) were also upregulated in both cell lines partially depleted for *ZIP11* (Supplementary Figure S7A and Supplementary Table S4)*.* Additional senescence genes (i.e. *CXCL1*, *CXCL2*, *CSF2*, and *ANKRD1*) presented a small increase in HeLa cells transduced with shRNA2, with minor changes in shRNA1 cells (Supplementary Table S4).

Changes were also detected in the expression of the metalloprotective transcription factor, MTF1, and the target gene *METALLOTHIONEIN*, *MT1A* (Supplementary Figures S7B,C and Supplementary Table S4). However, the RNA-seq analyses showed that the members of the network of Zn exporters and importers, *ZnTs1-8,10* and *ZIPs1-13*, do not present any significant changes in their expression (Supplementary Table S4). Only the gene encoding *ZIP14* has a significant and consistent decrease in its expression in both KD HeLa cells. The data suggest that the cells are unable to cope with the nuclear Zn stress produced by the KD of ZIP11, and potentially the impaired growth may be due to a senescent state and decrease in cell cycle progression rate induced by nuclear Zn dysregulation. Distinctive GO categories between shRNA-1 and shRNA-2 are shown in Supplementary Figure S8.

### Expression of Exogenous ZIP11 Rescues the Proliferation Defect, Restores Nuclear Zn Levels, and Confers Resistance to Elevated Levels of the Metal

**FIGURE 5:**
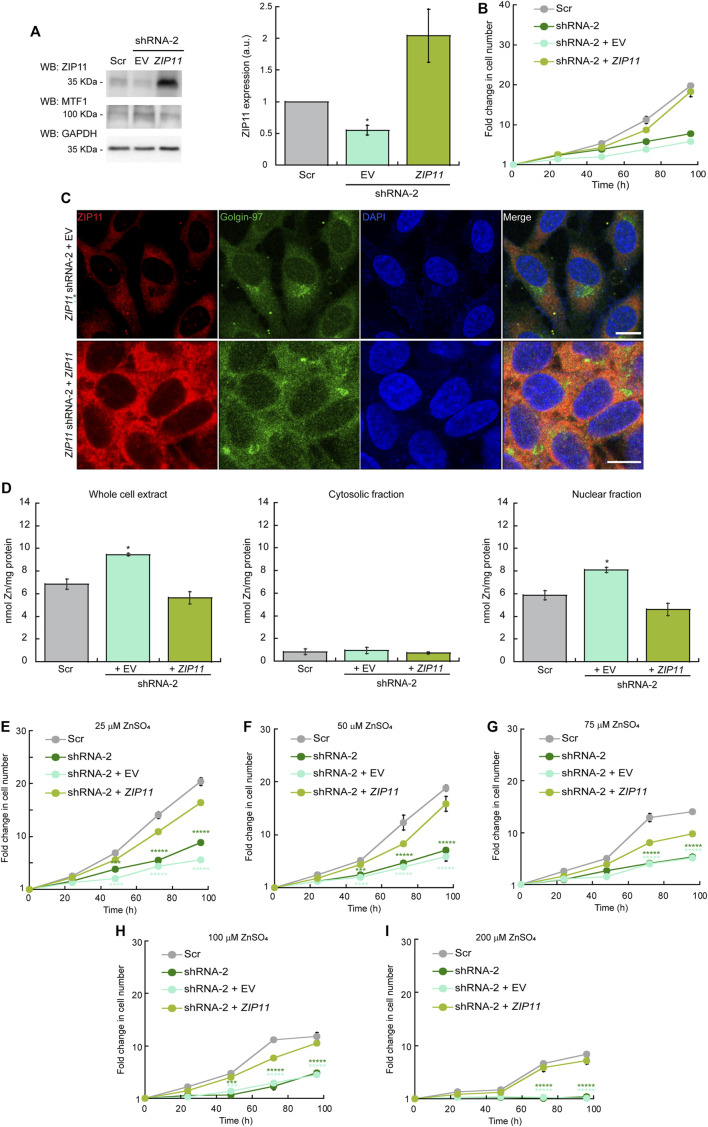
(Continued).

To determine whether the lack of ZIP11 and increase in nuclear Zn levels are responsible for the growth defect observed in KD cells, we reintroduced the *ZIP11* gene using a standard protocol of viral transduction and generated clones stably expressing the protein (Figure 5). Cells transduced with an empty vector (EV) were used as controls. For the reconstitution experiments, we used cells expressing the shRNA-2 for *ZIP11* KD, as this shRNA recognizes the UTR of the transporter gene. Expression of exogenous ZIP11 was confirmed by immunoblot (Figure 5A). Consistent with our RNA-Seq and gene expression profiles, under normal metal conditions, the expression of MTF1 protein was elevated in cells KD for ZIP11 and restored to basal levels in cells expressing exogenous *ZIP11* gene (Figure 5A). The proliferation defect detected in *ZIP11* KD cells was rescued upon expression of WT ZIP11 as shown by cell counting assays (Figure 5B). Confocal microscopy analyses showed an increase in the staining of ZIP11 in a perinuclear and cytosolic punctuated pattern upon reintroducing the gene to the KD cells (Figure 5C). Importantly, the cells expressing ZIP11 also presented a concentration of nuclear Zn similar to the levels of control cells (Figure 5D By contrast, the cells transduced with the EV maintained the proliferation defect, reduced levels of the protein in the perinuclear area and maintained elevated levels of the metal in the nucleus (Figures 5B–D). We then asked whether reintroduction of the gene would also restore cell resistance to extracellular Zn stress. The cells were grown under increasing concentrations of ZnSO_4_ and proliferation was determined by cell counting assays. Figures 5E–I show that cells expressing the recombinant transporter are less sensitive to Zn stress and can grow at a rate similar to control cells. As expected, the cells transduced with EV were sensitive to extracellular Zn stress as non-transduced cells. Thus, the data supports a role for ZIP11 in maintaining nuclear Zn homeostasis.

### Overexpression of Exogenous ZIP11 Exacerbates the Growth of HeLa Cells and Provides Elevated Resistance to External Zn Stress

**FIGURE 6:**
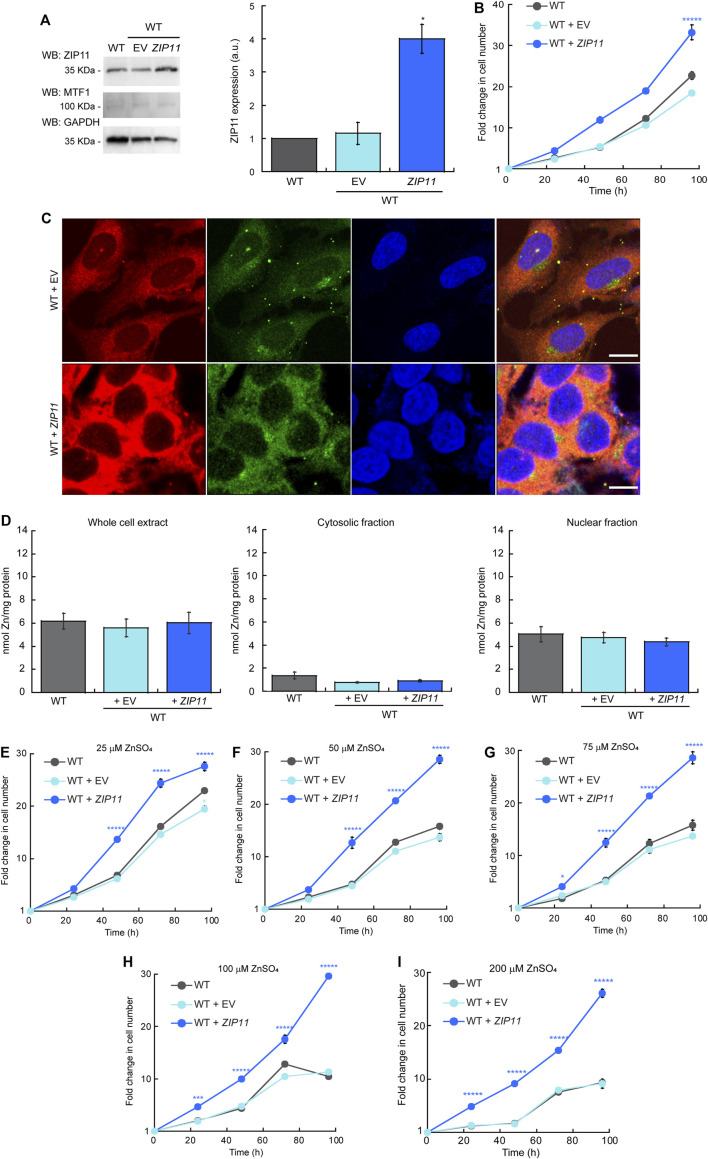
(Continued).

To further understand the effect of ZIP11 in the proliferation and metal resistance of HeLa cells, we performed overexpression experiments where WT cells were transduced with and stably expressed either the *ZIP11* gene or the EV as a control (Figure 6). Evaluation of ZIP11 by western blot shows that cells transduced with the vector encoding the *ZIP11* gene expressed a significantly larger amount of the transporter compared to non-transduced EV-infected control cells (Figure 6A). In this case, the levels of MTF1 protein remained constant in the three cell lines tested (Figure 6A). Cell proliferation assays revealed that overexpression of *ZIP11* in WT cells enhanced proliferation (Figure 6B). Confocal microscopy analyses showed an increased perinuclear and cytosolic punctuated staining for ZIP11 (Figure 6C), similar to the observed pattern of reconstitution experiments (Figure 5C), though the total, cytosolic, and nuclear levels of Zn remained stable and similar to control cells (Figure 6C). Importantly, the cells overexpressing ZIP11 were significantly more resistant to elevated levels of Zn (up to 200 µM) supplemented in the culture media than control cells (Figures 6D–H). The data corroborates a function for ZIP11 in maintaining nuclear Zn homeostasis to enable appropriate gene regulation and cell growth.

### ZIP11 KD Impairs the Migration and Invasive Properties of HeLa Cells

**FIGURE 7:**
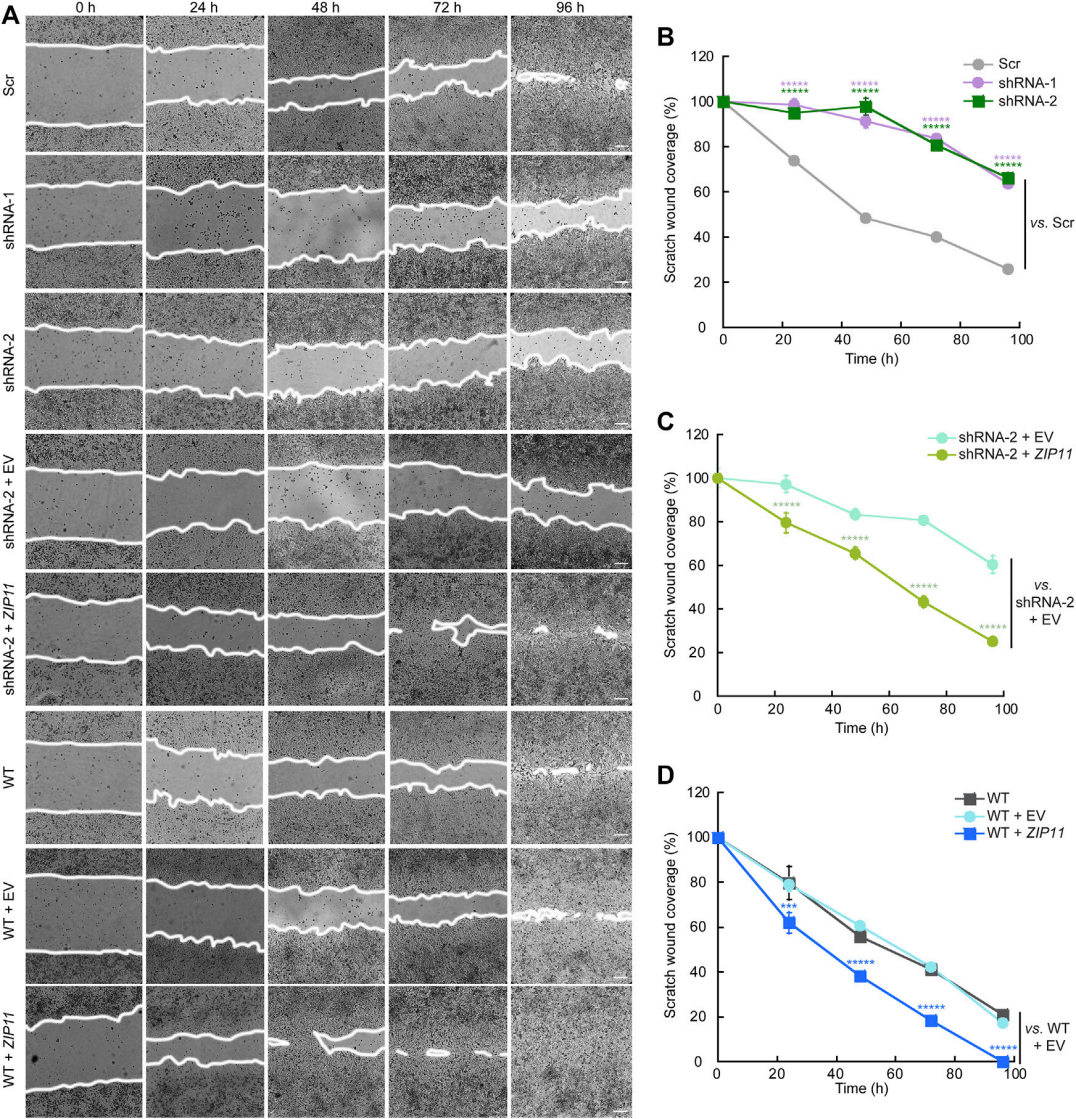
*ZIP11* is required for directional migration of HeLA cells. **(A)** Representative light microscopy images of the wound healing assay of HeLA cells in which *ZIP11* was KD or overexpressed. Time 0 represents confluent monolayer wounds at 0 h and wounds were monitored until the monolayers of WT cells overexpressing *ZIP11* became fully closed 96 h after scratching the monolayer. Images are representative of three independent biological replicates. Scale bar: 100 μm. **(B**–**D)** Quantification of the area of migration over time shown in **(A)**. **(B)** HeLa cells expressing Scr and both shRNAs against *ZIP11*. **(C)** Data for the reconstitution of phenotype of *ZIP11* KD cells. **(D)** Migration data of non-transduced WT HeLa cells and those overexpressing ZIP11 or the empty vector (EV). Data represents the means ± SE of three independent biological replicates imaged. *****p < 0.00001 relative to the samples indicated in the plot.

Cancer cells have several hallmarks and biological functions that promote EMT and metastasis. Thus far, we have evidence showing that ZIP11 is required for the growth of HeLa cells. Therefore, we utilized two functional assays to assess the contributions of ZIP11 to the carcinogenic phenotype of these cells. First, we performed a wound-healing assay, wherein a confluent cell monolayer is scratched and the time and extent of cell migration to close the wound was determined. Figure 7 shows a time course of representative light microscopy images of the wound-healing assay for WT HeLa cells, cells transduced with Scr, shRNA-1, and shRNA-2, and cells reconstituted and overexpressing *ZIP11* and the EV. Time 0 h indicates the moment when the wound is performed, and subsequent pictures are representative of subsequent time points (taken every 24 h) where the cells were monitored to determine the time needed for the wound to close (Figure 7A). Quantification of the area migrated over time showed that the rate of migration of *ZIP11* KD cells into the wound was reduced compared to Scr controls (Figure 7B). This deficient migration phenotype was reverted by reintroducing the exogenous *ZIP11* gene into the KD cells (Figure 7C), and was enhanced in WT cells overexpressing ZIP11, as these cells fully covered the wound 1 day earlier than the rest of the cells (Figure 7D). Thus, directional migration induced by a wound closure is impaired with ZIP11 KD and conversely enhanced by ZIP11 overexpression.

**FIGURE 8:**
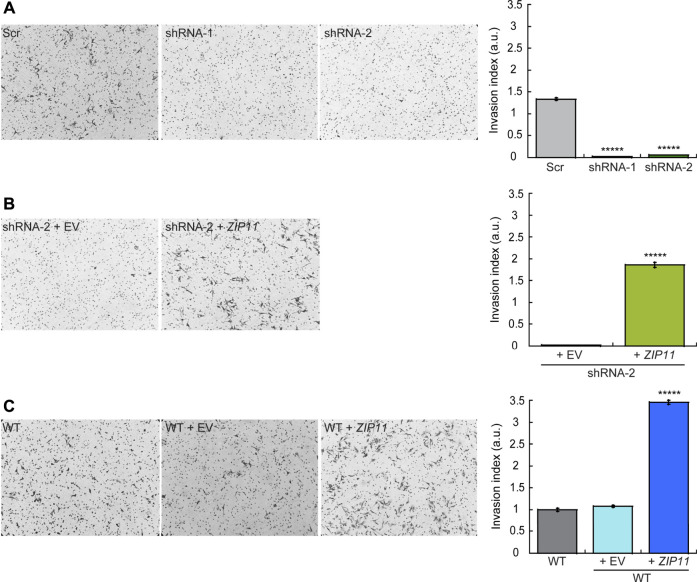
ZIP11 is required for HeLa cells invasion through Matrigel. Representative light microscopy images (left panels) and quantification (right panels) of Matrigel invasion assay at 24 h for HeLa cells in which ZIP11 was KD **(A)**, reconstituted in the shRNA2 strain **(B),** or overexpressed in WT cells **(C)**. The data show the means ± SE of three independent biological replicates imaged and are expressed as the percentage of invading cells compared to the control shown in the plots. *****p < 0.00001.

To further investigate the functional consequences of decreasing the expression of ZIP11 in HeLa cells, we also studied their invasive properties through Matrigel, a basement membrane extract. In this experiment, cells were seeded on the top of a polycarbonate membrane with 8 µm pores covered with Matrigel. This model allows invasive cells to cross and invade the opposite side of the membrane, which are then fixed and stained (Figure 8). To prevent cell proliferation, the cells were pre-treated with AraC before performing the invasion assays (Olea-Flores et al., 2019; Lacombe et al., 2021). Consistent with the migration results, we found that after 24 h of culture the *ZIP11* KD cells were unable to cross the matrix and the membrane, while control cells could colonize the other side of the membrane (Figure 8A). As expected, reconstitution of *ZIP11* gene in the shRNA-2 KD cells recovered the invasive phenotype (Figure 8B), and overexpression of the transporter in WT cells exacerbated the effect (Figure 8C). On average, the cells overexpressing *ZIP11* had a 3.5-fold increase in number of cells migrating across the Matrigel and the membrane pores compared to the control cells. Together, these data indicate that the transporter, and potentially nuclear Zn homeostasis, are important players in the development of the migratory and invasive phenotype in cancer cells. The fact that *ZIP11* KD cells have impaired migration and invasion of the Matrigel supports the idea of a potential dormancy or senescent state triggered by nuclear Zn dysregulation. Conversely, the increase in migration and invasion through Matrigel when ZIP11 is overexpressed supports the idea of a role for this transporter in promoting aggressive cancer phenotypes observed in cervical cancer patients (Figure 1 and Supplementary Figure S1).

### Alterations in Cell Cycle Progression and in Functional Senescence Markers Reflect a Potential Senescent State of the Cells KD for *ZIP11*

**FIGURE 9:**
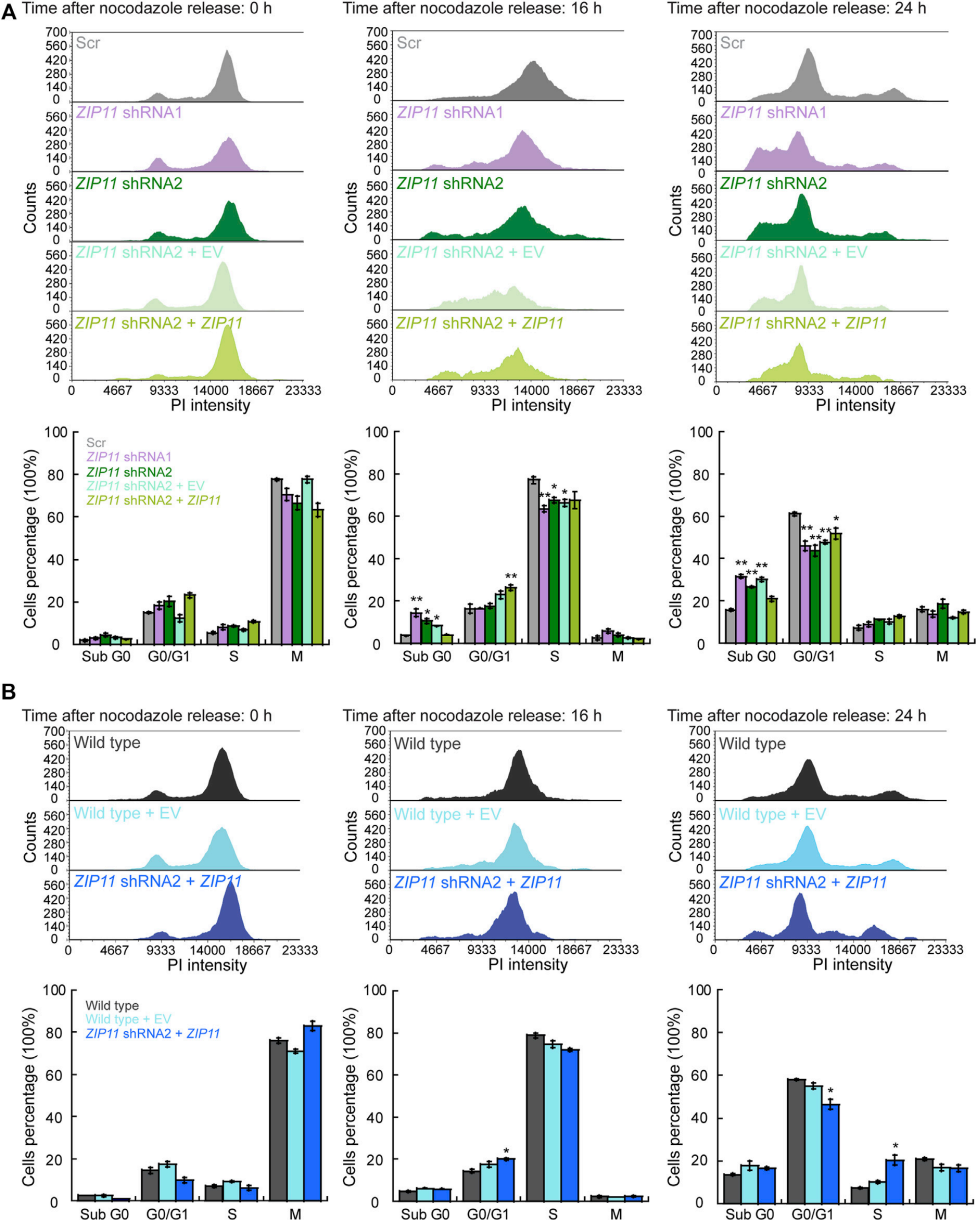
Knockdown of ZIP11 alters cell cycle progression. Representative histograms of cell cycle progression (top panels) and percentage of cells in each cell cycle phase (bottom panel). **(A)** HeLa cells transduced with Scr, *ZIP11* shRNA1 or shRNA2 and reconstituted with an empty vector or *ZIP11* gene. **(B)** Wild type HeLa cells transduced with an empty vector or overexpressing *ZIP11.* Plots show cells arrested in mitosis with nocodazole at the time of release (0 h), and after 16 and 24 h post-release. The data are representative of three independent biological experiments. *p < 0.05; **p < 0.01.

Increasing evidence points to a correlation between DNA damage, cellular senescence, and mitochondrial dysfunction as hallmarks of aging and the onset of various age-related pathologies, such as cancer (Reviewed by (Chapman et al., 2019; Gudmundsrud et al., 2021)). To better understand the growth defect and decreased mobility and invasion properties of HeLa cells KD for *ZIP11,* we tested for changes in cell cycle progression and metabolic changes of the senescence marker β-galactosidase and mitochondrial membrane potential. Our data show that ZIP11 contributes to proliferation and to re-entry into the cell cycle following release from a nocodazole-induced mitotic block, as KD HeLa cells present a delayed progression of the cycle and accumulate in sub G0 after 24 h of arrest (Figure 9A). The arrest in sub G0 phase was rescued by reintroduction of exogenous *ZIP11* into the KD cells. Consistent with the enhanced proliferation effect observed in wild type HeLa cells overexpressing the transporter, we detected a small but significant increase of cells in S phase compared to control cells that were largely in G0/G1 stage (Figure 9B). This data suggests that ZIP11 contributes, at least in part, to successful transition through cell cycle, which is also consistent with the gene expression changes shown in our RNA-seq analyses.

**FIGURE 10:**
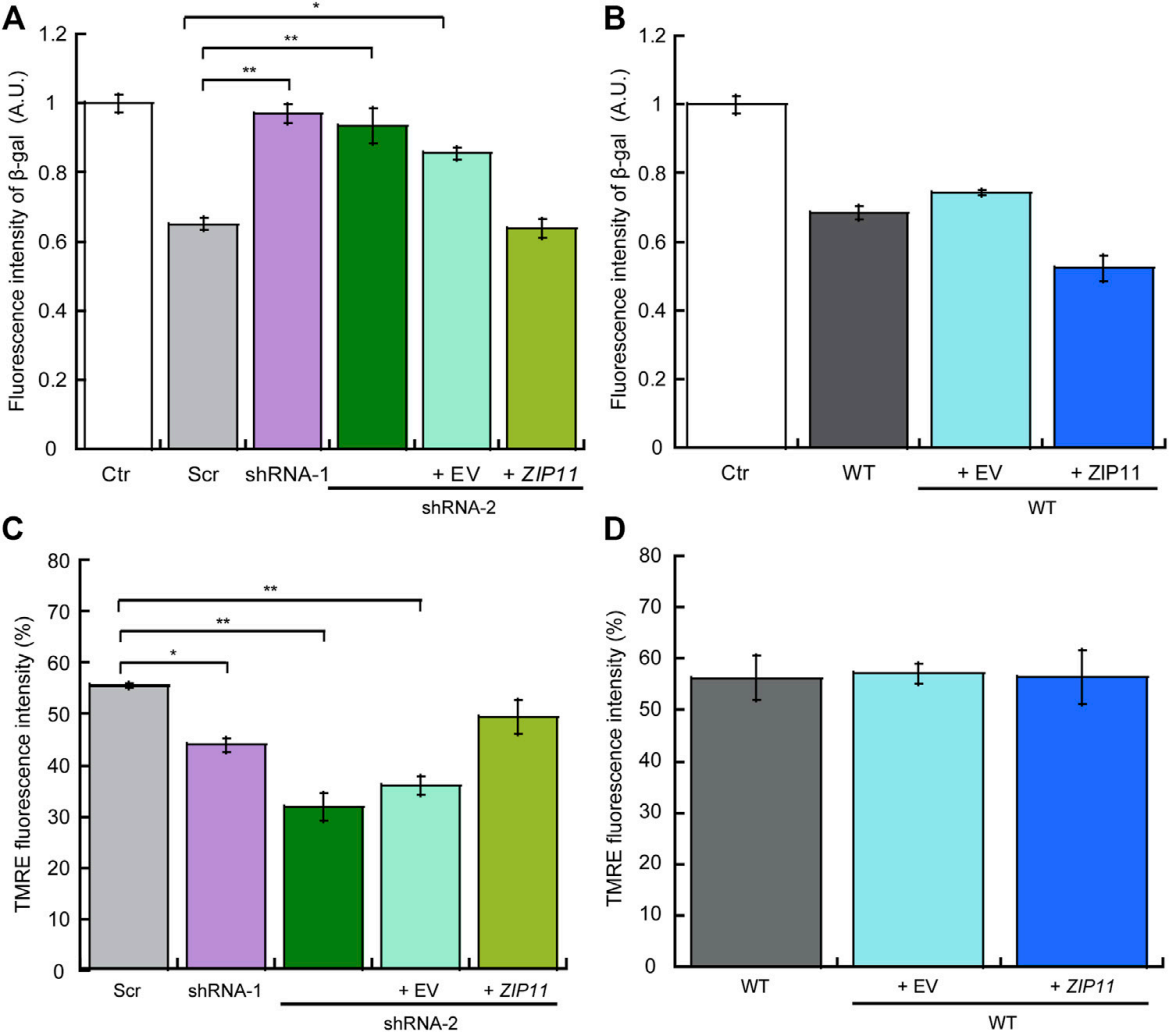
Knockdown of ZIP11 induces a senescent state which correlates with decreased mitochondrial potential in HeLa cells. Detection of cellular senescence *via* activation of β-galactosidase hydrolysis, a marker for senescent cells, in Scr, *ZIP11* KD cells and reconstituted with exogenous *ZIP11* **(A)** or wild type cells over expressing the transporter **(B)**. WT HeLa cells treated with 5 mM Palbociclib were used as a positive control for senescence (Ctr). Data was normalized to Palbociclib treated cells. Mitochondrial membrane potential was measured by staining cells KD for ZIP11, reconstituted **(C)** or overexpressing **(D)** the exogenous gene with 200 nM TMRE and the percentage of fluorescence intensity of three independent biological replicates was plotted. Data show means ± SE of three independent biological replicates imaged. *p < 0.05, **p < 0.01 relative to control.

To further provide insight into whether *ZIP11* KD induces a dormant or senescent state in the cells, we performed a classic functional assay of β-galactosidase activity to evaluate senescence in cells. The CellEvent Senescence Green assay relies on a fluorescent probe that contains two galactoside fractions which are targets for β-galactosidase (β-gal), a marker for senescent cells. The activation of the hydrolase activity of β-gal occurs in lysosomes under acidic pH and converts β-galactosides into monosaccharides which remains in the cell and emit a fluorescent signal. Figure 10A shows that *ZIP11* KD cells present an increase in the activation of β-gal which is similar to senescent control cells, which was reverted by reintroducing the transporter. Interestingly, a small but not significant decrease in β-gal activity was detected for wild type HeLa cells overexpressing ZIP11 (Figure 10B). Finally, we investigated the mitochondrial potential of HeLa cells KD and overexpressing ZIP11 using a TMRE assay as a proxy measure of their metabolic state. TMRE is a positively-charged, permeable dye that enters the cells and accumulates in active mitochondria, as this organelle presents a relatively high negative charge. When cells have depolarized or their mitochondria are inactive, a decrease in mitochondrial membrane potential consequently impairs the internalization of the TMRE dye. The data show that HeLa cells partially depleted of ZIP11 have a significant decrease in the incorporation of TMRE into the mitochondria compared to control cells, as indicated by a decrease in the intensity of the fluorescent signal of TMRE (Figure 10C). This decrease in mitochondrial function and potential can be restored upon reintroduction of the *ZIP11* gene, but not when the cells are transduced with the EV plasmid (Figure 10C). No significant changes were detected in TMRE incorporation into the mitochondria of WT cells overexpressing the ZIP11 transporter (Figure 10D). Together, the data suggest that nuclear control of Zn homeostasis by ZIP11 contributes to cell cycle progression and establishment of carcinogenic properties in HeLa cells.

## Discussion

Cancer development and progression encompasses metabolic changes that rely on the bioavailability of transition metals, like Zn, to promote cell growth and development of metastatic properties. In this work, we provide evidence that ZIP11 is a Zn transporter is located in the perinuclear area and in small vesicles partially associated to Golgi that may contribute to the maintenance of metal homeostasis in the nuclei. We determined the functional significance of ZIP11 expression by decreasing the levels of this transporter using a stable shRNA KD strategy in proliferating HeLa cells. We found that dysregulation of nuclear Zn levels produced by the ZIP11 KD resulted in a delay in cell cycle progression and a potential senescent state in the cells that may be related to DNA damage, as suggested by the alterations in expression of cell cycle and some senescence genes (Pospelova et al., 2009). RNA-Seq analyses also showed that angiogenic, EMT-related, and apoptotic genes were dysregulated. In terms of expression of additional Zn transporters, ZIPs and ZNTs, we detected no significant changes on their gene expression, which suggested that ZIP11 KD cells fail to compensate for the nuclear metal stress induced by ZIP11 malfunction. In the context of cancer patients, altered levels of Zn have been considered an indicator of tumor burden and disease progression (Prasad and Kucuk, 2002; Prasad et al., 2009). Zn defects promote the expression of the tumor suppressor p53 and affect the DNA binding capacity of several transcription factors, including p53, the nuclear factor κB (NFκB), and AP-1 in various models of cancer (Ho and Ames, 2002; Yan et al., 2008; Ho and Song, 2009). Zn is also proposed to repress tumor growth by decreasing angiogenesis, and by promoting the expression of inflammatory cytokines and apoptotic genes in cancer cells (Boehm et al., 1998; Prasad et al., 2009). Studies in murine models demonstrated that Zn treatment increases resistance against tumor growth and decreases the occurrence of spontaneous lung tumors in mice undergoing anti-carcinogenic therapies (Singh et al., 1992; Satoh et al., 1993). Consistent with these findings, Zn deficiency in rats enhances the proliferation and expression of cell cycle markers and promotes development of tumors derived from esophageal cells stimulated with the tumorigenic agent N-nitrosomethylbezylamine. This effect can be reduced by dietary Zn supplementation by inducing apoptosis (Wangu et al., 1996). Although existing evidence supports an anti-cancer role for Zn, there is still lack of understanding of the direct and indirect mechanisms by which Zn impacts cancer cells biology.

*ZIP11* KD cells not only accumulated nuclear Zn and failed to proliferate, but also showed a decrease in migration and invasive properties, as well as a reduction in mitochondrial membrane potential, and increased β-gal activity, which supports the hypothesis of a potential senescent state (Passos et al., 2006; Passos and von Zglinicki, 2012; Gudmundsrud et al., 2021). These phenotypes were reverted by reintroducing the WT transporter into the *ZIP11* KD cells. Consistent with these data, enhanced proliferation, migration, and invasive features were detected in WT HeLa cells overexpressing ZIP11. Interestingly, the levels of nuclear Zn in cells overexpressing the transporter were similar to those in WT control cells. This phenotype can be partially explained by the fact that no free Zn is found in the nucleus, and that nuclear Zn-binding proteins may have higher affinity to the ion and require the metal for proper function. Therefore, it is plausible that these proteins will not release the Zn even if the transporter expression increase. Importantly, confocal microscopy analyses showed elevated levels of ZIP11 in cytosolic vesicles, and some partially associated to Golgi. It is plausible that additional components of such vesicles contribute to the enhanced carcinogenic phenotype observed in cells overexpressing the transporters. The nature of such vesicles, their constituents and biological relevance remain to be characterized. Taken together, these data indicate that ZIP11 is essential for the proliferation and development of carcinogenic properties of the cervical cancer model, HeLa cells. This transporter may play a relevant role in the regulation of gene expression in the HeLa cell cancer model, which is in agreement with the correlation of a deleterious effect observed in cervical cancer patients that present elevated levels of ZIP11.

It is well known that senescence is a biological process that occurs in response to various stress stimuli under normal and pathological conditions. For instance, senescence can occur as a consequence of oncogene activation, chromatin and nuclear alterations, and oxidative stress (Kuilman et al., 2010). Senescence was also proposed to be a process that prevents cell replication when DNA damage occurs, and it is an efficient way to prevent cancer development and tumor progression (Campisi, 2001a; Campisi, 2001b). Interestingly, Zn has been shown to have a positive effect on DNA repair, which would prevent cancer development derived from DNA damage (Reviewed by (Yildiz et al., 2019)). Senescence is also known to suppress cancer by stopping the growth of pre-malignant cells, and it has been shown to be an important component for wound healing as well (Demaria et al., 2014). Experiments using a murine model in which senescent cells can be visualized and removed showed that senescent fibroblasts and endothelial cells appear very early in response to a cutaneous wound and promote the healing of the wound *via* the platelet-derived growth factor AA pathway (Demaria et al., 2014). From our observations in *ZIP11* KD HeLa cells, we proposed that the potential senescent phenotype resulting from nuclear Zn dysbalance impairs malignant cell mobilization. This rationale may also apply to the phenotype observed in the migratory properties of the model presented here. In conclusion, we propose a novel mechanism whereby elevated levels of Zn in the nuclei of cells lacking *ZIP11* is a contributing stress factor that impairs cell growth and other events associated with cancer cell biology (migration and invasion) by inducing a senescent state. This work highlights the importance of ZIP11, an understudied metal transporter, in cancer development and progression, and provides a foundation for future mechanistic and drug development studies that may target ZIP11 in patients affected by this disease.

## Data Availability

The datasets presented in this study can be found in online repositories. The names of the repository/repositories and accession number(s) can be found in the article/Supplementary Material. RNA-seq datasets are available at GEO. The accession number is: GSE198411

## References

[B1] Angireddy R. Chowdhury A. R. Zielonka J. Ruthel G. Kalyanaraman B. Avadhani N. G. (2020). Alcohol-induced CYP2E1, Mitochondrial Dynamics and Retrograde Signaling in Human Hepatic 3D Organoids. Free Radic. Biol. Med. 159, 1–14. 10.1016/j.freeradbiomed.2020.06.030 32738395 PMC13285058

[B2] Antala S. Dempski R. E. (2012). The Human ZIP4 Transporter Has Two Distinct Binding Affinities and Mediates Transport of Multiple Transition Metals. Biochemistry 51, 963–973. 10.1021/bi201553p 22242765

[B3] Audic S. Claverie J.-M. (1997). The Significance of Digital Gene Expression Profiles. Genome Res. 7, 986–995. 10.1101/gr.7.10.986 9331369

[B4] Barresi V. Valenti G. Spampinato G. Musso N. Castorina S. Rizzarelli E. (2018). Transcriptome Analysis Reveals an Altered Expression Profile of Zinc Transporters in Colorectal Cancer. J. Cell Biochem. 119, 9707–9719. 10.1002/jcb.27285 30129075

[B5] Bendl J. Stourac J. Salanda O. Pavelka A. Wieben E. D. Zendulka J. (2014). PredictSNP: Robust and Accurate Consensus Classifier for Prediction of Disease-Related Mutations. PLoS Comput. Biol. 10, e1003440. 10.1371/journal.pcbi.1003440 24453961 PMC3894168

[B6] Bin B.-H. Fukada T. Hosaka T. Yamasaki S. Ohashi W. Hojyo S. (2011). Biochemical Characterization of Human ZIP13 Protein. J. Biol. Chem. 286, 40255–40265. 10.1074/jbc.m111.256784 21917916 PMC3220551

[B7] Boehm T. O'Reilly M. S. M. Keough K. Shiloach J. Shapiro R. Folkman J. (1998). Zinc-binding of Endostatin Is Essential for its Antiangiogenic Activity. Biochem. biophysical Res. Commun. 252, 190–194. 10.1006/bbrc.1998.9617 9813168

[B8] Bradford M. M. (1976). A Rapid and Sensitive Method for the Quantitation of Microgram Quantities of Protein Utilizing the Principle of Protein-Dye Binding. Anal. Biochem. 72, 248–254. 10.1016/0003-2697(76)90527-3 942051

[B9] Broun E. R. Greist A. Tricot G. Hoffman R. (1990). Excessive Zinc Ingestion. Jama 264, 1441–1443. 10.1001/jama.1990.03450110087033 2094240

[B10] Campisi J. (2001). Cellular Senescence as a Tumor-Suppressor Mechanism. Trends Cell Biol. 11, S27–S31. 10.1016/s0962-8924(01)82148-6 11684439

[B11] Campisi J. (2001). Cellular Senescence, Aging and Cancer. TheScientificWorldJournal 1, 65. 10.1100/tsw.2001.23.106 PMC6084413 30147535

[B12] Chapman J. Fielder E. Passos J. F. (2019). Mitochondrial Dysfunction and Cell Senescence: Deciphering a Complex Relationship. FEBS Lett. 593, 1566–1579. 10.1002/1873-3468.13498 31211858

[B13] Chowanadisai W. Graham D. M. Keen C. L. Rucker R. B. Messerli M. A. (2013). Neurulation and Neurite Extension Require the Zinc Transporter ZIP12 ( Slc39a12 ). Proc. Natl. Acad. Sci. U.S.A. 110, 9903–9908. 10.1073/pnas.1222142110 23716681 PMC3683776

[B14] Chowdhury A. R. Zielonka J. Kalyanaraman B. Hartley R. C. Murphy M. P. Avadhani N. G. (2020). Mitochondria-targeted Paraquat and Metformin Mediate ROS Production to Induce Multiple Pathways of Retrograde Signaling: A Dose-dependent Phenomenon. Redox Biol. 36, 101606. 10.1016/j.redox.2020.101606 32604037 PMC7327929

[B15] Collins F. S. Guyer M. S. Chakravarti A. (1997). Variations on a Theme: Cataloging Human DNA Sequence Variation. Science 278, 1580–1581. 10.1126/science.278.5343.1580 9411782

[B16] Colvin R. A. Bush A. I. Volitakis I. Fontaine C. P. Thomas D. Kikuchi K. (2008). Insights into Zn2+homeostasis in Neurons from Experimental and Modeling Studies. Am. J. Physiology-Cell Physiology 294, C726–C742. 10.1152/ajpcell.00541.2007 18184873

[B17] Demaria M. Ohtani N. Youssef S. A. Rodier F. Toussaint W. Mitchell J. R. (2014). An Essential Role for Senescent Cells in Optimal Wound Healing through Secretion of PDGF-AA. Dev. cell 31, 722–733. 10.1016/j.devcel.2014.11.012 25499914 PMC4349629

[B18] Dempski R. E. (2012). The Cation Selectivity of the ZIP Transporters. Curr. Top. Membr. 69, 221–245. 10.1016/b978-0-12-394390-3.00009-4 23046653

[B19] Devirgiliis C. Zalewski P. D. Perozzi G. Murgia C. (2007). Zinc Fluxes and Zinc Transporter Genes in Chronic Diseases. Mutat. Research/Fundamental Mol. Mech. Mutagen. 622, 84–93. 10.1016/j.mrfmmm.2007.01.013 17374385

[B20] Dufner-Beattie J. Langmade S. J. Wang F. Eide D. Andrews G. K. (2003). Structure, Function, and Regulation of a Subfamily of Mouse Zinc Transporter Genes. J. Biol. Chem. 278, 50142–50150. 10.1074/jbc.m304163200 14525987

[B21] Dufner-Beattie J. Wang F. Kuo Y.-M. Gitschier J. Eide D. Andrews G. K. (2003). The Acrodermatitis Enteropathica Gene ZIP4 Encodes a Tissue-specific, Zinc-Regulated Zinc Transporter in Mice. J. Biol. Chem. 278, 33474–33481. 10.1074/jbc.m305000200 12801924

[B22] Eide D. J. (2006). Zinc Transporters and the Cellular Trafficking of Zinc. Biochimica Biophysica Acta (BBA) - Mol. Cell Res. 1763, 711–722. 10.1016/j.bbamcr.2006.03.005 16675045

[B23] Fischer P. W. Giroux A. L'Abbé M. R. (1981). The Effect of Dietary Zinc on Intestinal Copper Absorption. Am. J. Clin. Nutr. 34, 1670–1675. 10.1093/ajcn/34.9.1670 7282591

[B24] Fujishiro H. Yano Y. Takada Y. Tanihara M. Himeno S. (2012). Roles of ZIP8, ZIP14, and DMT1 in Transport of Cadmium and Manganese in Mouse Kidney Proximal Tubule Cells. Metallomics 4, 700–708. 10.1039/c2mt20024d 22534978

[B25] Gaither L. A. Eide D. J. (2001). Eukaryotic Zinc Transporters and Their Regulation. Biometals 14, 251–270. 10.1023/a:1012988914300 11831460

[B26] Gaither L. A. Eide D. J. (2000). Functional Expression of the Human hZIP2 Zinc Transporter. J. Biol. Chem. 275, 5560–5564. 10.1074/jbc.275.8.5560 10681536

[B27] Gaither L. A. Eide D. J. (2001). The Human ZIP1 Transporter Mediates Zinc Uptake in Human K562 Erythroleukemia Cells. J. Biol. Chem. 276, 22258–22264. 10.1074/jbc.m101772200 11301334

[B28] Gao J. Zhao N. Knutson M. D. Enns C. A. (2008). The Hereditary Hemochromatosis Protein, HFE, Inhibits Iron Uptake via Down-Regulation of Zip14 in HepG2 Cells. J. Biol. Chem. 283, 21462–21468. 10.1074/jbc.m803150200 18524764 PMC2490774

[B29] Girijashanker K. He L. Soleimani M. Reed J. M. Li H. Liu Z. (2008). Slc39a14 Gene Encodes ZIP14, a Metal/bicarbonate Symporter: Similarities to the ZIP8 Transporter. Mol. Pharmacol. 73, 1413–1423. 10.1124/mol.107.043588 18270315 PMC2753210

[B30] Gordon S. J. V. Fenker D. E. Vest K. E. Padilla-Benavides T. (2019). Manganese Influx and Expression of ZIP8 Is Essential in Primary Myoblasts and Contributes to Activation of SOD_2_ . Metallomics 11 (6), 1140–1153. 10.1039/c8mt00348c 31086870 PMC6584035

[B31] Gordon S. J. V. Xiao Y. Paskavitz A. L. Navarro-Tito N. Navea J. G. Padilla-Benavides T. (2019). Atomic Absorbance Spectroscopy to Measure Intracellular Zinc Pools in Mammalian Cells. J. Vis. Exp. 1, 1. 10.3791/59519 31157776

[B32] Gudmundsrud R. Skjånes T. H. Gilmour B. C. Caponio D. Lautrup S. Fang E. F. (2021). Crosstalk Among DNA Damage, Mitochondrial Dysfunction, Impaired Mitophagy, Stem Cell Attrition, and Senescence in the Accelerated Ageing Disorder Werner Syndrome. Cytogenet Genome Res. 161, 297–304. 10.1159/000516386 34433164 PMC8491497

[B33] Haase H. Rink L. (2014). Zinc Signals and Immune Function. Biofactors 40, 27–40. 10.1002/biof.1114 23804522

[B34] Hambidge M. (2000). Human Zinc Deficiency. J. Nutr. 130, 1344S–1349S. 10.1093/jn/130.5.1344s 10801941

[B35] Ho E. Ames B. N. (2002). Low Intracellular Zinc Induces Oxidative DNA Damage, Disrupts P53, NFκB, and AP1 DNA Binding, and Affects DNA Repair in a Rat Glioma Cell Line. Proc. Natl. Acad. Sci. U.S.A. 99, 16770–16775. 10.1073/pnas.222679399 12481036 PMC139219

[B36] Ho E. Song Y. (2009). Zinc and Prostatic Cancer. Curr. Opin. Clin. Nutr. Metabolic Care 12, 640–645. 10.1097/mco.0b013e32833106ee PMC4142760 19684515

[B37] Hojyo S. Fukada T. Shimoda S. Ohashi W. Bin B.-H. Koseki H. (2011). The Zinc Transporter SLC39A14/ZIP14 Controls G-Protein Coupled Receptor-Mediated Signaling Required for Systemic Growth. PloS one 6, e18059. 10.1371/journal.pone.0018059 21445361 PMC3062567

[B38] Hu J. (2021). Toward Unzipping the ZIP Metal Transporters: Structure, Evolution, and Implications on Drug Discovery against Cancer. Febs J. 288, 5805–5825. 10.1111/febs.15658 33296542

[B39] Huang L. Kirschke C. P. (2007). A Di-leucine Sorting Signal in ZIP1 (SLC39A1) Mediates Endocytosis of the Protein. FEBS J. 274, 3986–3997. 10.1111/j.1742-4658.2007.05933.x 17635580

[B40] Itzhak D. N. Tyanova S. Cox J. Borner G. H. (2016). Global, Quantitative and Dynamic Mapping of Protein Subcellular Localization. eLife 5, 1. 10.7554/eLife.16950 PMC4959882 27278775

[B41] Jenkitkasemwong S. Wang C.-Y. Coffey R. Zhang W. Chan A. Biel T. (2015). SLC39A14 Is Required for the Development of Hepatocellular Iron Overload in Murine Models of Hereditary Hemochromatosis. Cell metab. 22, 138–150. 10.1016/j.cmet.2015.05.002 26028554 PMC4497937

[B42] Jenkitkasemwong S. Wang C.-Y. Mackenzie B. Knutson M. D. (2012). Physiologic Implications of Metal-Ion Transport by ZIP14 and ZIP8. Biometals 25, 643–655. 10.1007/s10534-012-9526-x 22318508 PMC4598647

[B43] Jeong J. Eide D. J. (2013). The SLC39 Family of Zinc Transporters. Mol. aspects Med. 34, 612–619. 10.1016/j.mam.2012.05.011 23506894 PMC3602797

[B44] Kaida A. Sawai N. Sakaguchi K. Miura M. (2011). Fluorescence Kinetics in HeLa Cells after Treatment with Cell Cycle Arrest Inducers Visualized with Fucci (Fluorescent Ubiquitination-Based Cell Cycle Indicator). Cell. Biol. Int. 35, 359–363. 10.1042/cbi20100643 21231917

[B45] Kambe T. Hashimoto A. Fujimoto S. (2014). Current Understanding of ZIP and ZnT Zinc Transporters in Human Health and Diseases. Cell. Mol. Life Sci. 71, 3281–3295. 10.1007/s00018-014-1617-0 24710731 PMC11113243

[B46] Kambe T. Tsuji T. Hashimoto A. Itsumura N. (2015). The Physiological, Biochemical, and Molecular Roles of Zinc Transporters in Zinc Homeostasis and Metabolism. Physiol. Rev. 95, 749–784. 10.1152/physrev.00035.2014 26084690

[B47] Kamphans T. Sabri P Fau - Zhu N. Zhu N Fau - Heinrich V. Heinrich V Fau - Mundlos S. Mundlos S Fau - Robinson P. N. Robinson Pn Fau - Parkhomchuk D. (2013). Filtering for Compound Heterozygous Sequence Variants in Non-consanguineous Pedigrees. Plos One 8 (8), e70151. 10.1371/journal.pone.0070151 23940540 PMC3734130

[B48] Kang X. Chen R. Zhang J. Li G. Dai P.-G. Chen C. (2015). Expression Profile Analysis of Zinc Transporters (ZIP4, ZIP9, ZIP11, ZnT9) in Gliomas and Their Correlation with IDH1 Mutation Status. Asian Pac. J. Cancer Prev. 16, 3355–3360. 10.7314/apjcp.2015.16.8.3355 25921144

[B49] Kelleher S. L. Lönnerdal B. (2003). Zn Transporter Levels and Localization Change throughout Lactation in Rat Mammary Gland and Are Regulated by Zn in Mammary Cells. J. Nutr. 133, 3378–3385. 10.1093/jn/133.11.3378 14608047

[B50] Kelleher S. L. Velasquez V. Croxford T. P. McCormick N. H. Lopez V. MacDavid J. (2012). Mapping the Zinc-Transporting System in Mammary Cells: Molecular Analysis Reveals a Phenotype-dependent Zinc-Transporting Network during Lactation. J. Cell. Physiol. 227, 1761–1770. 10.1002/jcp.22900 21702047 PMC3207005

[B51] Kim D. Langmead B. Salzberg S. L. (2015). HISAT: a Fast Spliced Aligner with Low Memory Requirements. Nat. Methods 12, 357–360. 10.1038/nmeth.3317 25751142 PMC4655817

[B52] Kim D. Xiao Y. Karchere-Sun R. Richmond E. Ricker H. M. Leonardi A. (2020). Atmospheric Processing of Anthropogenic Combustion Particles: Effects of Acid Media and Solar Flux on the Iron Mobility from Fly Ash. ACS Earth Space Chem. 4, 750–761. 10.1021/acsearthspacechem.0c00057

[B53] Krezel A. Maret W. (2006). Zinc-buffering Capacity of a Eukaryotic Cell at Physiological pZn. J. Biol. Inorg. Chem. 11, 1049–1062. 10.1007/s00775-006-0150-5 16924557

[B54] Kuilman T. Michaloglou C. Mooi W. J. Peeper D. S. (2010). The Essence of Senescence: Figure 1. Genes Dev. 24, 2463–2479. 10.1101/gad.1971610 21078816 PMC2975923

[B55] Lacombe M.-L. Lamarche F. De Wever O. Padilla-Benavides T. Carlson A. Khan I. (2021). The Mitochondrially-Localized Nucleoside Diphosphate Kinase D (NME4) Is a Novel Metastasis Suppressor. BMC Biol. 19, 228. 10.1186/s12915-021-01155-5 34674701 PMC8529772

[B56] Langmead B. Salzberg S. L. (2012). Fast Gapped-Read Alignment with Bowtie 2. Nat. Methods 9, 357–359. 10.1038/nmeth.1923 22388286 PMC3322381

[B57] Li B. Dewey C. N. (2011). RSEM: Accurate Transcript Quantification from RNA-Seq Data with or without a Reference Genome. BMC Bioinforma. 12, 323. 10.1186/1471-2105-12-323 PMC3163565 21816040

[B58] Lichten L. A. Cousins R. J. (2009). Mammalian Zinc Transporters: Nutritional and Physiologic Regulation. Annu. Rev. Nutr. 29, 153–176. 10.1146/annurev-nutr-033009-083312 19400752

[B59] Lichten L. A. Ryu M.-S. Guo L. Embury J. Cousins R. J. (2011). MTF-1-mediated Repression of the Zinc Transporter Zip10 Is Alleviated by Zinc Restriction. PloS one 6, e21526. 10.1371/journal.pone.0021526 21738690 PMC3124522

[B60] Lin W. Chai J. Love J. Fu D. (2010). Selective Electrodiffusion of Zinc Ions in a Zrt-, Irt-like Protein, ZIPB*. J. Biol. Chem. 285, 39013–39020. 10.1074/jbc.m110.180620 20876577 PMC2998139

[B61] Liu Z. Li H. Soleimani M. Girijashanker K. Reed J. M. He L. (2008). Cd2+ versus Zn2+ Uptake by the ZIP8 HCO3--dependent Symporter: Kinetics, Electrogenicity and Trafficking. Biochem. biophysical Res. Commun. 365, 814–820. 10.1016/j.bbrc.2007.11.067 PMC2212618 18037372

[B62] Liuzzi J. P. Aydemir F. Nam H. Knutson M. D. Cousins R. J. (2006). Zip14 (Slc39a14) Mediates Non-transferrin-bound Iron Uptake into Cells. Proc. Natl. Acad. Sci. U.S.A. 103, 13612–13617. 10.1073/pnas.0606424103 16950869 PMC1564235

[B63] Liuzzi J. P. Bobo J. A. Lichten L. A. Samuelson D. A. Cousins R. J. (2004). Responsive Transporter Genes within the Murine Intestinal-Pancreatic axis Form a Basis of Zinc Homeostasis. Proc. Natl. Acad. Sci. U.S.A. 101, 14355–14360. 10.1073/pnas.0406216101 15381762 PMC521973

[B64] Livak K. J. Schmittgen T. D. (2001). Analysis of Relative Gene Expression Data Using Real-Time Quantitative PCR and the 2−ΔΔCT Method. Methods 25, 402–408. 10.1006/meth.2001.1262 11846609

[B65] Love M. I. Huber W. Anders S. (2014). Moderated Estimation of Fold Change and Dispersion for RNA-Seq Data with DESeq2. Genome Biol. 15, 550. 10.1186/s13059-014-0550-8 25516281 PMC4302049

[B66] Mao X. Kim B.-E. Wang F. Eide D. J. Petris M. J. (2007). A Histidine-Rich Cluster Mediates the Ubiquitination and Degradation of the Human Zinc Transporter, hZIP4, and Protects against Zinc Cytotoxicity. J. Biol. Chem. 282, 6992–7000. 10.1074/jbc.m610552200 17202136

[B67] Maret W. Sandstead H. H. (2006). Zinc Requirements and the Risks and Benefits of Zinc Supplementation. J. Trace Elem. Med. Biol. 20, 3–18. 10.1016/j.jtemb.2006.01.006 16632171

[B68] Martin A. B. Aydemir T. B. Guthrie G. J. Samuelson D. A. Chang S.-M. Cousins R. J. (2013). Gastric and Colonic Zinc Transporter ZIP11 (Slc39a11) in Mice Responds to Dietary Zinc and Exhibits Nuclear Localization. J. Nutr. 143, 1882–1888. 10.3945/jn.113.184457 24089422 PMC3827636

[B69] Noren Hooten N. Evans M. K. (2017). Techniques to Induce and Quantify Cellular Senescence. J. Vis. Exp. 1, 55533. 10.3791/55533 PMC5565152 28518126

[B70] Ogiso T. Ogawa N. Miura T. (1979). Inhibitory Effect of High Dietary Zinc on Copper Absorption in Rats. II. Binding of Copper and Zinc to Cytosol Proteins in the Intestinal Mucosa. Chem. Pharm. Bull. 27, 515–521. 10.1248/cpb.27.515 445686

[B71] Olea-Flores M. Zuñiga-Eulogio M. Tacuba-Saavedra A. Bueno-Salgado M. Sánchez-Carvajal A. Vargas-Santiago Y. (2019). Leptin Promotes Expression of EMT-Related Transcription Factors and Invasion in a Src and FAK-dependent Pathway in MCF10A Mammary Epithelial Cells. Cells 8, 1133. 10.3390/cells8101133 PMC6829404 31554180

[B72] Outten C. E. O'Halloran a. T. V. (2001). Femtomolar Sensitivity of Metalloregulatory Proteins Controlling Zinc Homeostasis. Science 292, 2488–2492. 10.1126/science.1060331 11397910

[B73] Palmiter R. D. Findley S. D. (1995). Cloning and Functional Characterization of a Mammalian Zinc Transporter that Confers Resistance to Zinc. EMBO J. 14, 639–649. 10.1002/j.1460-2075.1995.tb07042.x 7882967 PMC398127

[B74] Paskavitz A. L. Quintana J. Cangussu D. Tavera-Montañez C. Xiao Y. Ortiz-Miranda S. (2018). Differential Expression of Zinc Transporters Accompanies the Differentiation of C2C12 Myoblasts. J. Trace Elem. Med. Biol. 49, 27–34. 10.1016/j.jtemb.2018.04.024 29895369 PMC6082398

[B75] Passos J. F. von Zglinicki T. (2012). Mitochondrial Dysfunction and Cell Senescence - Skin Deep into Mammalian Aging. Aging 4, 74–75. 10.18632/aging.100432 22337807 PMC3314168

[B76] Passos J. F. Zglinicki T. v. Saretzki G. (2006). Mitochondrial Dysfunction and Cell Senescence: Cause or Consequence? Rejuvenation Res. 9, 64–68. 10.1089/rej.2006.9.64 16608398

[B77] Pertea M. Pertea G. M. Antonescu C. M. Chang T.-C. Mendell J. T. Salzberg S. L. (2015). StringTie Enables Improved Reconstruction of a Transcriptome from RNA-Seq Reads. Nat. Biotechnol. 33, 290–295. 10.1038/nbt.3122 25690850 PMC4643835

[B78] Pinilla-Tenas J. J. Sparkman B. K. Shawki A. Illing A. C. Mitchell C. J. Zhao N. (2011). Zip14 Is a Complex Broad-Scope Metal-Ion Transporter Whose Functional Properties Support Roles in the Cellular Uptake of Zinc and Nontransferrin-Bound Iron. Am. J. Physiology-Cell PhysiologyCell physiology 301, C862–C871. 10.1152/ajpcell.00479.2010 PMC3191563 21653899

[B79] Pospelova T. V. Demidenko Z. N. Bukreeva E. I. Pospelov V. A. Gudkov A. V. Blagosklonny M. V. (2009). Pseudo-DNA Damage Response in Senescent Cells. Cell Cycle 8, 4112–4118. 10.4161/cc.8.24.10215 19946210 PMC4970747

[B80] Prasad A. S. Beck F. W. J. Snell D. C. Kucuk O. (2009). Zinc in Cancer Prevention. Nutr. cancer 61, 879–887. 10.1080/01635580903285122 20155630

[B81] Prasad A. S. Kucuk O. (2002). Zinc in Cancer Prevention. Cancer Metastasis Rev. 21, 291–295. 10.1023/a:1021215111729 12549767

[B82] Qin Y. Dittmer P. J. Park J. G. Jansen K. B. Palmer A. E. (2011). Measuring Steady-State and Dynamic Endoplasmic Reticulum and Golgi Zn 2+ with Genetically Encoded Sensors. Proc. Natl. Acad. Sci. U.S.A. 108, 7351–7356. 10.1073/pnas.1015686108 21502528 PMC3088641

[B83] Rafi M. A. Coppola S Fau - Liu S. L. Liu Sl Fau - Rao H. Z. Rao Hz Fau - Wenger D. A. Wenger D. A. (2003). Disease-causing Mutations in Cis with the Common Arylsulfatase A Pseudodeficiency Allele Compound the Difficulties in Accurately Identifying Patients and Carriers of Metachromatic Leukodystrophy. Mol. Genet. Metab. 79 (2), 83–90. 10.1016/s1096-7192(03)00076-3 12809637

[B84] Risch N. Merikangas K. (1996). The Future of Genetic Studies of Complex Human Diseases. Science 273, 1516–1517. 10.1126/science.273.5281.1516 8801636

[B85] Rost B. Yachdav G. Liu J. (2004). The PredictProtein Server. Nucleic Acids Res. 32, W321–W326. 10.1093/nar/gkh377 15215403 PMC441515

[B86] Sandstead H. H. (2013). Human Zinc Deficiency: Discovery to Initial Translation. Adv. Nutr. 4, 76–81. 10.3945/an.112.003186 23319126 PMC3648742

[B87] Satoh M. Kondo Y. Mita M. Nakagawa I. Naganuma A. Imura N. (1993). Prevention of Carcinogenicity of Anticancer Drugs by Metallothionein Induction. Cancer Res. 53, 4767–4768. 8402657

[B88] Schindelin J. Arganda-Carreras I. Frise E. Kaynig V. Longair M. Pietzsch T. (2012). Fiji: an Open-Source Platform for Biological-Image Analysis. Nat. Methods 9, 676–682. 10.1038/nmeth.2019 22743772 PMC3855844

[B89] Sensi S. L. Canzoniero L. M. T. Yu S. P. Ying H. S. Koh J.-Y. Kerchner G. A. (1997). Measurement of Intracellular Free Zinc in Living Cortical Neurons: Routes of Entry. J. Neurosci. 17, 9554–9564. 10.1523/jneurosci.17-24-09554.1997 9391010 PMC6573416

[B90] Singh K. P. Zaidi S. I. A. Raisuddin S. Saxena A. K. Murthy R. C. Ray P. K. (1992). Effect of Zinc on Immune Functions and Host Resistance against Infection and Tumor Challenge. Immunopharmacol. Immunotoxicol. 14, 813–840. 10.3109/08923979209009237 1338205

[B91] Suzuki K. Bose P. Leong-Quong R. Y. Fujita D. J. Riabowol K. (2010). REAP: A Two Minute Cell Fractionation Method. BMC Res. Notes 3, 294. 10.1186/1756-0500-3-294 21067583 PMC2993727

[B92] Takeda A. Tamano H. (2009). Insight into Zinc Signaling from Dietary Zinc Deficiency. Brain Res. Rev. 62, 33–44. 10.1016/j.brainresrev.2009.09.003 19747942

[B93] Tavera-Montañez C. Hainer S. J. Cangussu D. Gordon S. J. V. Xiao Y. Reyes-Gutierrez P. (2019). The Classic Metal-Sensing Transcription Factor MTF1 Promotes Myogenesis in Response to Copper. Faseb J. 33, 14556–14574. 10.1096/fj.201901606R 31690123 PMC6894080

[B94] Taylor K. M. Hiscox S. Nicholson R. I. Hogstrand C. Kille P. (2012). Protein Kinase CK2 Triggers Cytosolic Zinc Signaling Pathways by Phosphorylation of Zinc Channel ZIP7. Sci. Signal 5, ra11. 10.1126/scisignal.2002585 22317921 PMC3428905

[B95] Taylor K. M. (2000). LIV-1 Breast Cancer Protein Belongs to New Family of Histidine-Rich Membrane Proteins with Potential to Control Intracellular Zn 2+ Homeostasis. IUBMB Life (International Union Biochem. Mol. Biol. Life) 49, 249–253. 10.1080/15216540050033087 10995024

[B96] Taylor K. M. Morgan H. E. Johnson A. Nicholson R. I. (2005). Structure-function Analysis of a Novel Member of the LIV-1 Subfamily of Zinc Transporters, ZIP14. FEBS Lett. 579, 427–432. 10.1016/j.febslet.2004.12.006 15642354

[B97] Taylor K. M. Nicholson R. I. (2003). The LZT Proteins; the LIV-1 Subfamily of Zinc Transporters. Biochimica Biophysica Acta (BBA) - Biomembr. 1611, 16–30. 10.1016/s0005-2736(03)00048-8 12659941

[B98] Thiers R. E. Vallee B. L. (1957). Distribution of Metals in Subcellular Fractions of Rat Liver. J. Biol. Chem. 226, 911–920. 10.1016/s0021-9258(18)70877-6 13438880

[B99] Trapnell C. Williams B. A. Pertea G. Mortazavi A. Kwan G. van Baren M. J. (2010). Transcript Assembly and Quantification by RNA-Seq Reveals Unannotated Transcripts and Isoform Switching during Cell Differentiation. Nat. Biotechnol. 28, 511–515. 10.1038/nbt.1621 20436464 PMC3146043

[B100] Vallee B. L. Falchuk K. H. (1993). The Biochemical Basis of Zinc Physiology. Physiol. Rev. 73, 79–118. 10.1152/physrev.1993.73.1.79 8419966

[B101] Vinkenborg J. L. Nicolson T. J. Bellomo E. A. Koay M. S. Rutter G. A. Merkx M. (2009). Genetically Encoded FRET Sensors to Monitor Intracellular Zn2+ Homeostasis. Nat. Methods 6, 737–740. 10.1038/nmeth.1368 19718032 PMC6101214

[B102] Wang F. Kim B.-E. Petris M. J. Eide D. J. (2004). The Mammalian Zip5 Protein Is a Zinc Transporter that Localizes to the Basolateral Surface of Polarized Cells. J. Biol. Chem. 279, 51433–51441. 10.1074/jbc.m408361200 15322118

[B103] Wangu Q.-S. Sabourin C. L. K. Wang H. Stoner G. D. (1996). Overexpression of Cyclin D1 and Cyclin E in N-Nitrosomethylbezylamine-Induced Rat Esophageal Tumorigenesis. Carcinogenesis 17, 1583–1588. 10.1093/carcin/17.8.1583 8761413

[B104] Weaver B. P. Dufner-Beattie J. Kambe T. Andrews G. K. (2007). Novel Zinc-Responsive Post-transcriptional Mechanisms Reciprocally Regulate Expression of the Mouse Slc39a4 and Slc39a5 Zinc Transporters (Zip4 and Zip5). Biol. Chem. 388, 1301–1312. 10.1515/bc.2007.149 18020946 PMC2376820

[B105] Wu F. Y. H. Wu C. W. (1987). Zinc in DNA Replication and Transcription. Annu. Rev. Nutr. 7, 251–272. 10.1146/annurev.nu.07.070187.001343 2440465

[B106] Wu L. Chaffee K. G. Parker A. S. Sicotte H. Petersen G. M. (2015). Zinc Transporter Genes and Urological Cancers: Integrated Analysis Suggests a Role for ZIP11 in Bladder Cancer. Tumor Biol. 36, 7431–7437. 10.1007/s13277-015-3459-2 PMC4607554 25900876

[B107] Yan M. Song Y. Wong C. P. Hardin K. Ho E. (2008). Zinc Deficiency Alters DNA Damage Response Genes in Normal Human Prostate Epithelial Cells. J. Nutr. 138, 667–673. 10.1093/jn/138.4.667 18356318 PMC4152237

[B108] Yildiz A. Kaya Y. Tanriverdi O. (2019). Effect of the Interaction between Selenium and Zinc on DNA Repair in Association with Cancer Prevention. J. Cancer Prev. 24, 146–154. 10.15430/jcp.2019.24.3.146 31624720 PMC6786808

[B109] Yu Y. Wu A. Zhang Z. Yan G. Zhang F. Zhang L. (2013). Characterization of the GufA Subfamily Member SLC39A11/Zip11 as a Zinc Transporter. J. Nutr. Biochem. 24, 1697–1708. 10.1016/j.jnutbio.2013.02.010 23643525

[B110] Zhang T. Liu J. Fellner M. Zhang C. Sui D. Hu J. (2017). Crystal Structures of a ZIP Zinc Transporter Reveal a Binuclear Metal Center in the Transport Pathway. Sci. Adv. 3, e1700344. 10.1126/sciadv.1700344 28875161 PMC5573306

[B111] Zhu B. Huo R. Zhi Q. Zhan M. Chen X. Hua Z.-C. (2021). Increased Expression of Zinc Transporter ZIP4, ZIP11, ZnT1, and ZnT6 Predicts Poor Prognosis in Pancreatic Cancer. J. Trace Elem. Med. Biol. 65, 126734. 10.1016/j.jtemb.2021.126734 33631610

