# Supplementary material for: ZIP11 Regulates Nuclear Zinc Homeostasis in HeLa Cells and Is Required for Proliferation and Establishment of the Carcinogenic Phenotype
Source: Front Cell Dev Biol. 2022 Jul 11;10:895433. doi: 10.3389/fcell.2022.895433 (PMC9309433; doi:10.3389/fcell.2022.895433)
Supplement: Supplementary file 1 [file DataSheet1.PDF]

Supplementary Material

ZIP11 regulates nuclear zinc homeostasis in HeLa cells and is required for proliferation and establishment of the carcinogenic phenotype.

Monserrat Olea-Flores[1,2], Julia Kan[1], Alyssa Carlson[1,3], Sabriya A. Syed[1], Cat McCann[1], Varsha Mondal[1], Cecily Szady[4], Heather M. Ricker[4], Amy McQueen[1], Juan G. Navea[4], Leslie A. Caromile[5], Teresita Padilla-Benavides[1]*

[1] Department of Molecular Biology and Biochemistry, Wesleyan University, Middletown, CT, 06459, USA

[2] Department of Biochemistry and Molecular Biotechnology, University of Massachusetts Chan Medical School, Worcester, MA, 01605, USA

[3] Current affiliation: Tisch Multiple Sclerosis Research Center of New York, New York, NY, 10019, USA

[4] Department of Chemistry, Skidmore College, Saratoga Springs, NY, USA

[5] Center for Vascular Biology, Department of Cell Biology, UCONN Health-Center, Farmington, Connecticut 06030-3501, United States

*Correspondence: T.P.-B. tpadillabena@wesleyan.edu

SUPPLEMENTARY FIGURES

SUPPLEMENTARY FIGURE 1

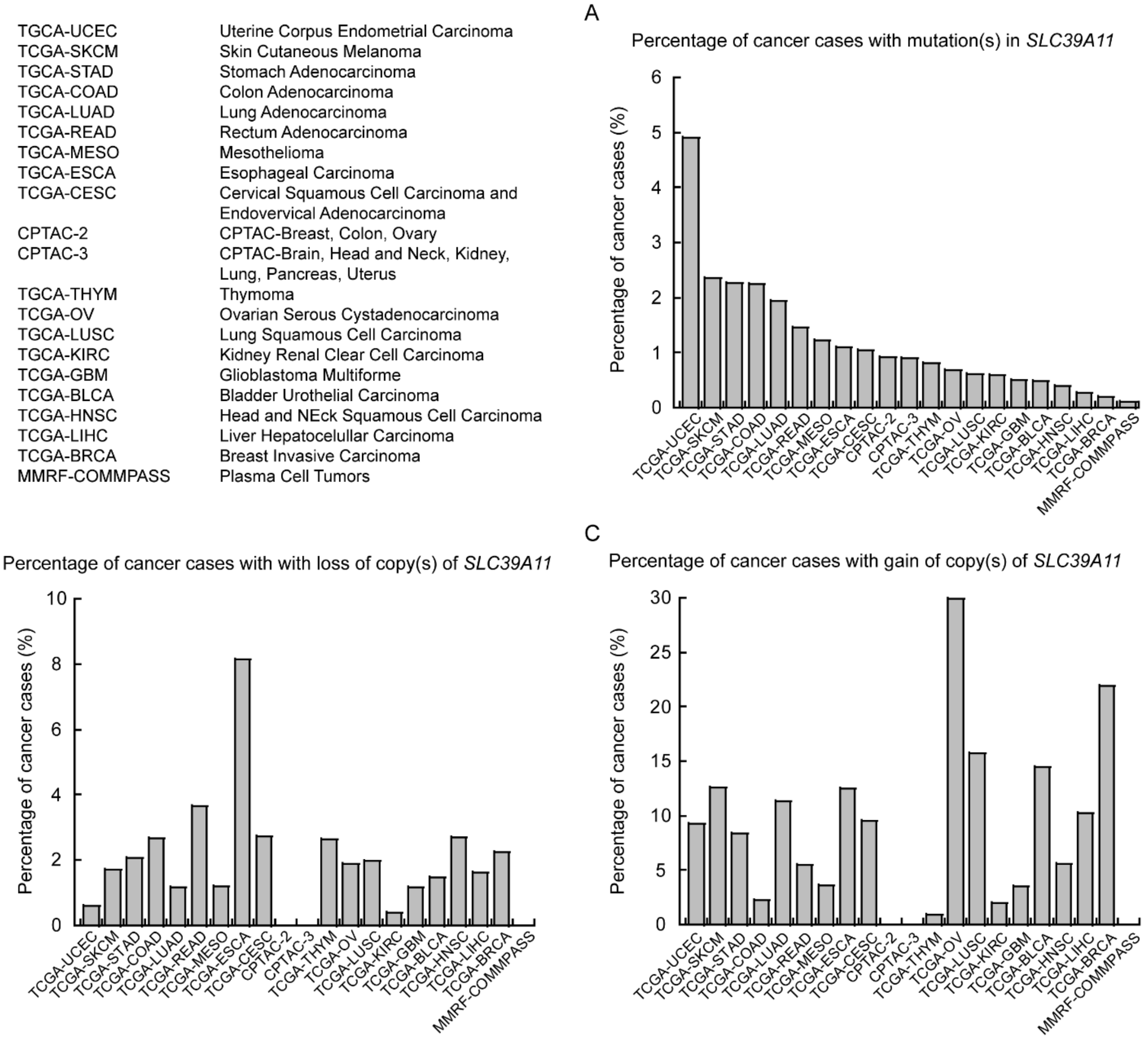

Supplementary Figure 1. Analyses of ZIP11 expression and mutation rate in patients obtained from TCGA database. (A) Incidence of mutations for ZIP11 gene occurs in cancer patients. Percentage of patients with loss **(B)** or gain **(C)** of function of the *ZIP11* gene.

SUPPLEMENTARY FIGURE 2

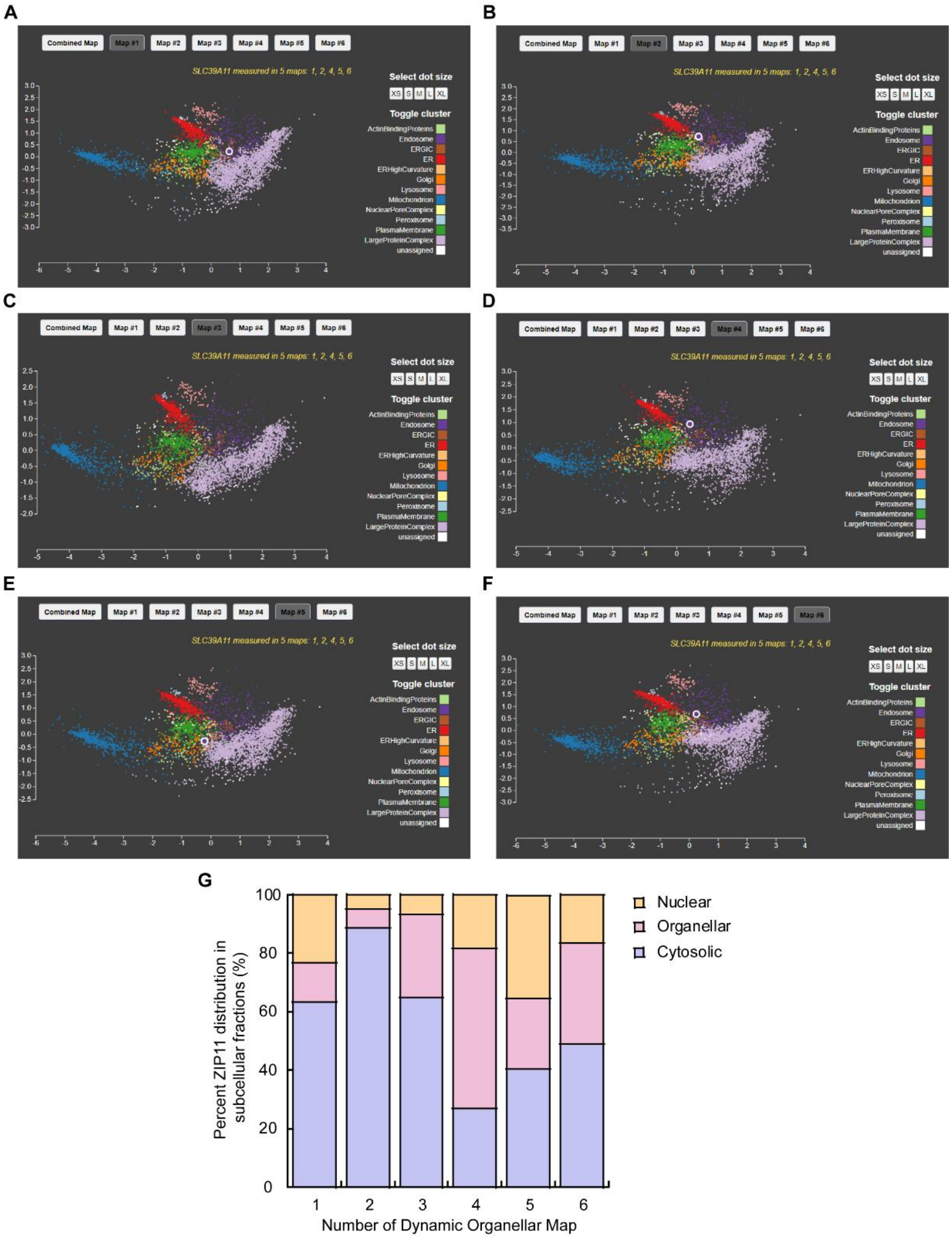

Supplementary Figure 2. Subcellular distribution of ZIP11 in HeLa cells determined by principal component analyses. A-F. Principal components analysis of six Dynamic Organellar Maps of ZIP11 generated by the interactive HeLa Spatial Proteome (http://mapofthecell.biochem.mpg.de/index.html [1]). The large white circle with a purple center on the Dynamic Organellar Maps indicates the cellular localization of ZIP11. **G.** The accompanying bar graphs also generated by the interactive HeLa Spatial Proteome, indicate the percent distribution of ZIP11 in the nucleus, cytosol, and organelles of the HeLa cells per Dynamic Organellar Map and the total percent for each map.

SUPPLEMENTARY FIGURE 3

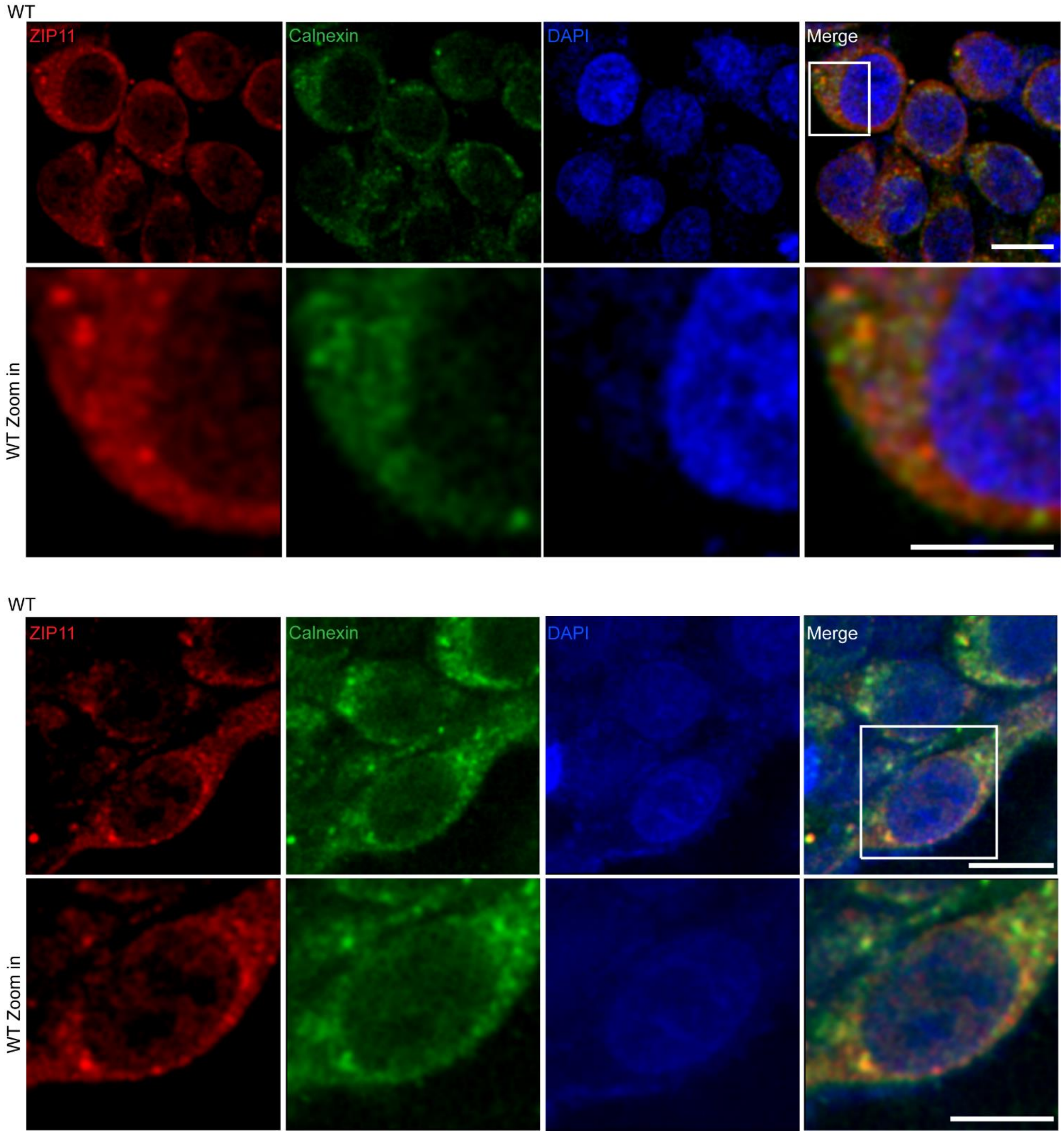

Supplementary Figure 3. ZIP11 and ER components do not colocalize in HeLa cells. Representative confocal images of two independent immunostainings showing a perinuclear and cytosolic punctuated pattern of expression of ZIP11 (red) in wild type HeLa cells. The anti-Calnexin (green) antibody was used as a marker of endoplasmic reticulum and nuclei was stained with DAPI (blue). White boxes are the zoomed-in areas presented for each of the replicates. Bar = 10 µm for low magnification images and 5 µm for zoom-in images.

SUPPLEMENTARY FIGURE 4

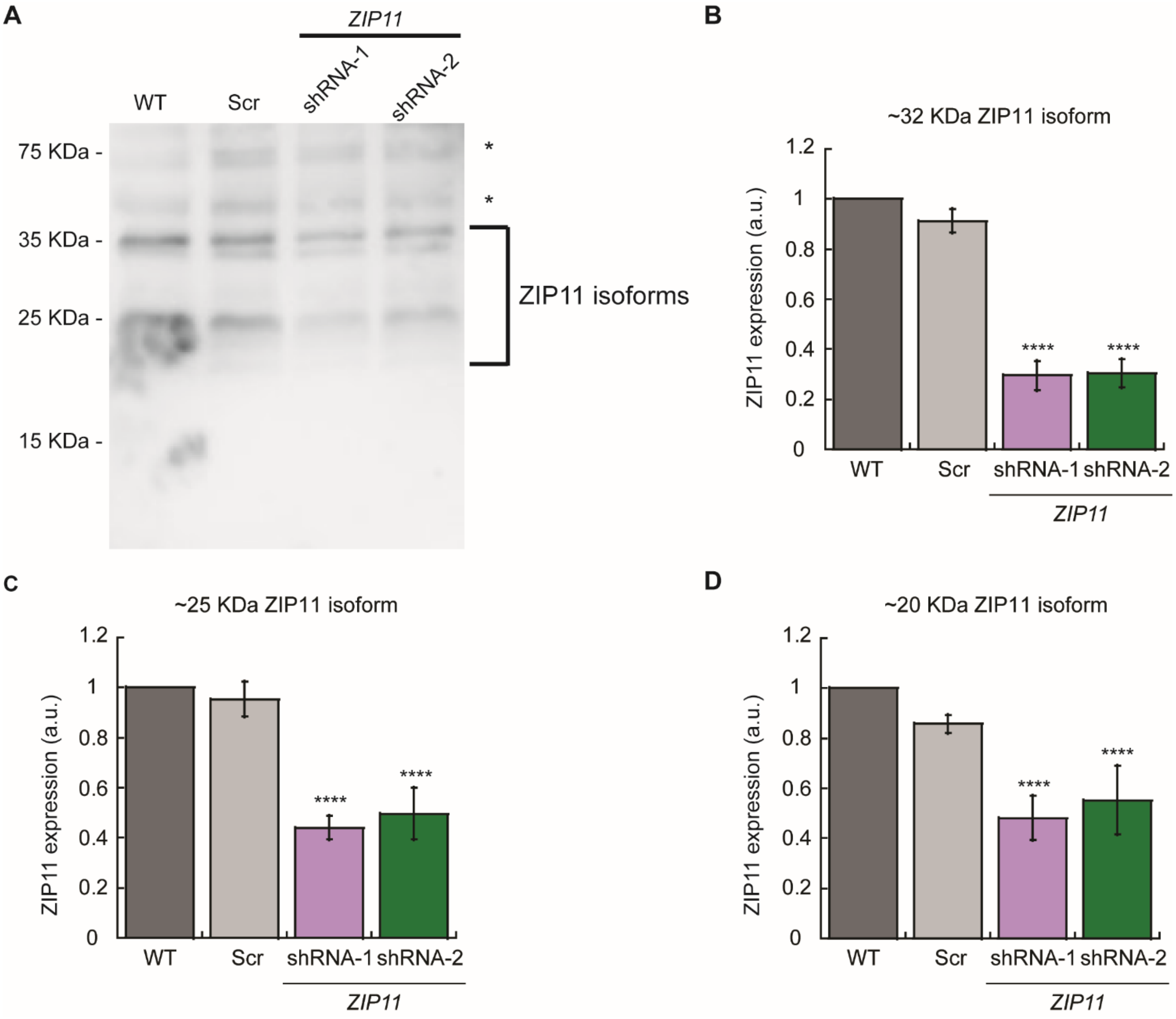

Supplementary Figure 3. ZIP11 isoforms in human cells. A. Full membrane of the representative immunoblot of *ZIP11* expression in HeLa proliferating cells shown in Fig. 2B. Several ZIP11 isoforms can be detected by the ZIP11 antibody used in this study. These isoforms are also KD by the shRNAs used for this study. Quantification of *ZIP11* levels of the protein levels ~32 KDa (**B**), ~25 KDa (**C**) and ~20 KDa (**D**) isoforms of ZIP11 in HeLa proliferating cells. Asterisks represent potential unspecific high molecular weight proteins or potential dimers of ZIP11. For all samples, data are the mean ± SE of three independent biological replicates. ****P < 0.0001.

SUPPLEMENTARY FIGURE 5

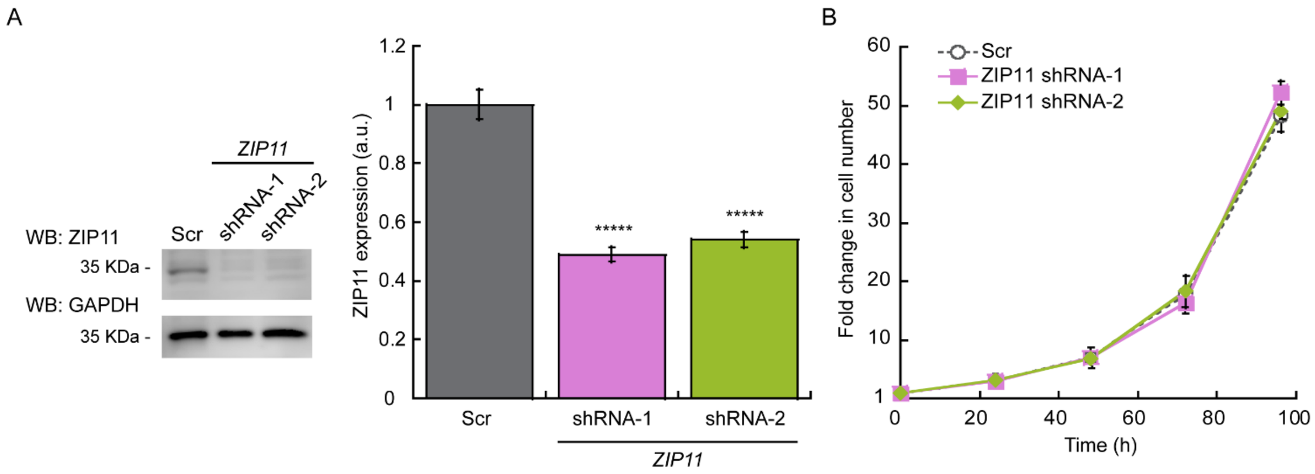

Supplementary Figure 5. ZIP11 KD does not affect the proliferation of HEK293T cells. A. Representative immunoblot (left) and quantification (right) of *ZIP11* levels in HEK293T proliferating cells. Immunoblots against GAPDH was used as loading control. Samples were compared to the corresponding Scr control. **C.** Cell counting assay of proliferating HEK293T cells transduced with scrambled shRNA (shRNA Scr), or *ZIP11* shRNAs. For all samples, data are the mean ± SE of three independent biological replicates. For all samples, data are the mean ± SE of three independent biological replicates. *****P < 0.00001.

SUPPLEMENTARY FIGURE 6

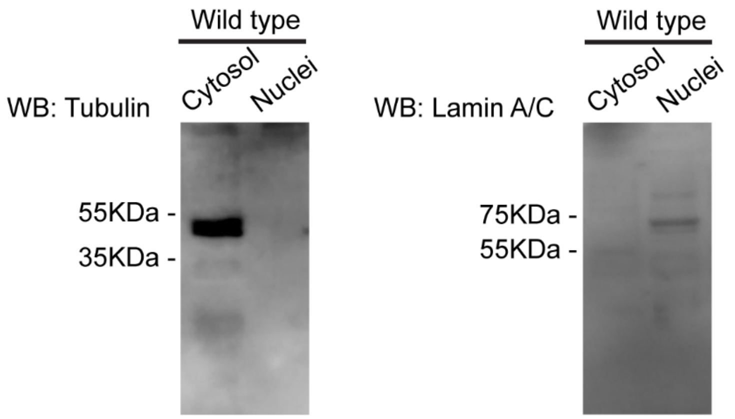

Supplemental Figure 6. Purity of subcellular fractions used in metal determinations. Representative western blot showing the purity of the subcellular fractions of wild type HeLa cells isolated using the REAP protocol [2; 3; 4]. Tubulin and Lamin A/C were used as controls to show the separation of cytoplasmic and nuclear fractions respectively.

SUPPLEMENTARY FIGURE 7

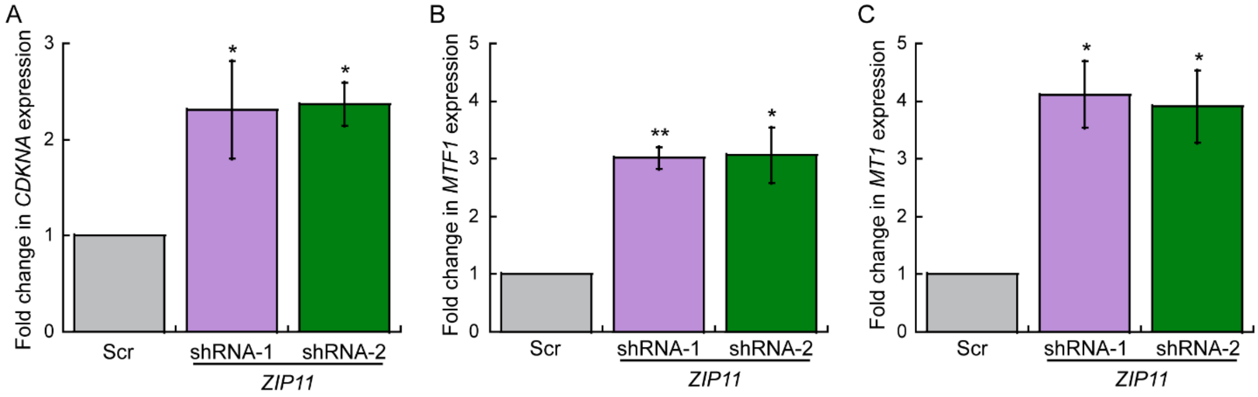

Supplemental Figure 7. Changes in the expression of senescence and metalloprotective genes dependent on *ZIP11* knockdown in HeLa cells. Steady state mRNA levels determined by qRT-PCR of representative up-regulated senescence gene [**(A)** *CDKNA* (p21)] and metalloprotective genes, *MTF1* and *MT1* genes (**B** and **C**, respectively). Data are the mean ± SE for three independent experiments. * $P < 0.05$, ** $P < 0.01$.

SUPPLEMENTARY FIGURE 8

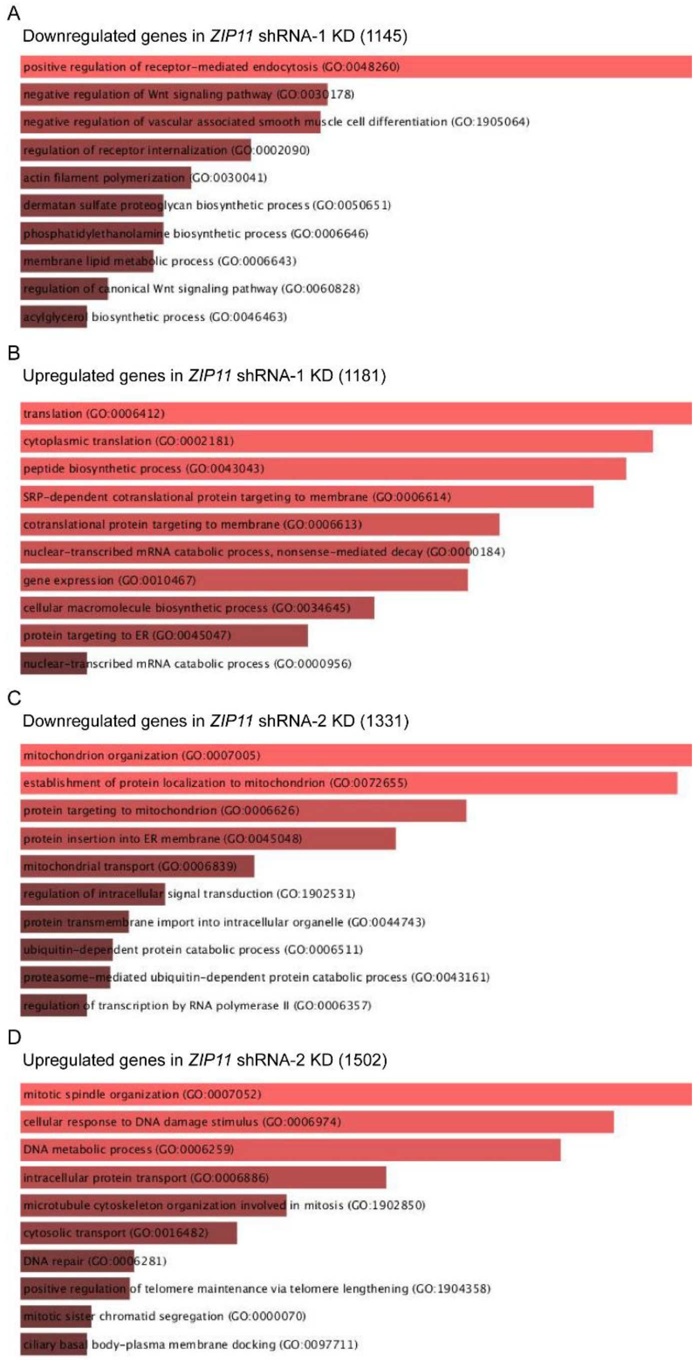

Supplemental Figure 8. GO term analyses representative of the DEG for individual *ZIP11* shRNA. GO term analysis of down-regulated (A) or up-regulated (B) genes for *ZIP11* shRNA-1 and down-regulated (C) or up-regulated (D) genes for *ZIP11* shRNA-2 in HeLa cells. Cut-off was set at 2.0 of the -log(adjusted P value). See Supp. Table 4 for the complete list of genes.

SUPPLEMENTARY TABLES

Supplementary table 1. Plasmids used in this study

Plasmid name	Specific information	Source	Use
Scr shRNA	MISSION® pLKO.1-puro Non-Target shRNA Control Plasmid DNA	Sigma-Aldrich SHC016	Control for shRNA KD
ZIP11 shRNA-1	5’- GAAGCCCAGATCAGTGGTAAT-3’	Sigma-Aldrich TRCN0000038367	KD *ZIP11* CDS
ZIP11 shRNA-2	5’-TCCTGATTGACTCTGATTATA-3’	Sigma-Aldrich TRCN0000434903	KD *ZIP11* UTR
pLV[Exp]-EGFP/Neo-EF1A>hSLC39A11[NM_001352692.2]	Human SLC39A11 ORF. Sequence available upon request	Vector Builder	Phenotype reconstitution
pLV[Exp]-EGFP/Neo-EF1A>empty vector	Empty vector	Vector Builder	Phenotype reconstitution

Supplementary table 2. Primers used in this study

Primer name	Forward sequence	Reverse sequence
CDKN2C	5’-AGACGCTTTCCGCATCAC-3’	5’-CTGAGCGGCATTAGCCCA-3’
CDK20	5’-CGGGCAAGAACGATATTG-3’	5’-TGGGTCAATTCCTTCTCT-3’
MOAP1	5’-GTCGATGAATGTCTGCAG-3’	5’-CGCCTAGACCAAGTCATT-3’
PPP2CA	5’-CCTCACGTTGGTGTCTAG-3’	5’-GTTCATGGCAATACTGTAC-3’
CDKN1A (p21)	5’-GACACCACTGGAGGGTGACT-3’	5’-CAGGTCCACATGGTCTTCCT-3’
MTF1	5’-ACCAAGAACAAATTCAGCAAGC-3’	5’-ACACTGAGGCCAATCTGCTG-3’
MT1	5’-CTCCTTGCCTCGAAATGGAC-3’	5’-GCATTTGCACTCTTTGCATTTG-3’
GAPDH	5’-GTCTCCTCTGACTTCAACAGCG-3’	5’-ACCACCCTGTTGCTGTAGCCAA-3’

Supplementary table 3. Comparison of Survival Curves

**Log-rank (Mantel-Cox) Test**	
Chi square	0.9845
df	1
P value	0.3211
P value summary	ns
Are the survival curves sig different?	No
**Gehan-Breslow-Wilcoxon Test**	
Chi square	0.0005788
df	1
P value	0.9808
P value summary	ns
Are the survival curves sig different?	No
**Median survival**	
High Expression	95.30
Low Expression	Undefined
**Hazard Ratio**	
Ratio	1.297
95% CI of ratio	0.7762 to 2.166

Supplementary references

[1] D.N. Itzhak, S. Tyanova, J. Cox, and G.H. Borner, Global, quantitative and dynamic mapping of protein subcellular localization. eLife 5 (2016).

[2] C. Tavera-Montanez, S.J. Hainer, D. Cangussu, S.J.V. Gordon, Y. Xiao, P. Reyes-Gutierrez, A.N. Imbalzano, J.G. Navea, T.G. Fazzio, and T. Padilla-Benavides, The classic metal-sensing transcription factor MTF1 promotes myogenesis in response to copper. Faseb J 33 (2019) 14556-14574.

[3] S.J.V. Gordon, Y. Xiao, A.L. Paskavitz, N. Navarro-Tito, J.G. Navea, and T. Padilla-Benavides, Atomic Absorbance Spectroscopy to Measure Intracellular Zinc Pools in Mammalian Cells. Journal of visualized experiments : JoVE (2019).

[4] K. Suzuki, P. Bose, R.Y. Leong-Quong, D.J. Fujita, and K. Riabowol, REAP: A two minute cell fractionation method. BMC research notes 3 (2010) 294.
